# Impact of Long-Term Organic and Mineral Fertilization on Rhizosphere Metabolites, Root–Microbial Interactions and Plant Health of Lettuce

**DOI:** 10.3389/fmicb.2020.597745

**Published:** 2021-01-13

**Authors:** Saskia Windisch, Loreen Sommermann, Doreen Babin, Soumitra Paul Chowdhury, Rita Grosch, Narges Moradtalab, Frank Walker, Birgit Höglinger, Abbas El-Hasan, Wolfgang Armbruster, Joseph Nesme, Søren Johannes Sørensen, Ingo Schellenberg, Jörg Geistlinger, Kornelia Smalla, Michael Rothballer, Uwe Ludewig, Günter Neumann

**Affiliations:** ^1^Department of Nutritional Crop Physiology, Institute of Crop Science, University of Hohenheim, Stuttgart, Germany; ^2^Institute of Bioanalytical Sciences (IBAS), Anhalt University of Applied Sciences, Bernburg, Germany; ^3^Institute for Epidemiology and Pathogen Diagnostics, Julius Kühn Institute – Federal Research Centre for Cultivated Plants, Braunschweig, Germany; ^4^Institute of Network Biology, Helmholtz Zentrum München, Neuherberg, Germany; ^5^Plant-Microbe Systems, Leibniz Institute of Vegetable and Ornamental Crops, Großbeeren, Germany; ^6^Central Chemical-Analytical Laboratory, Institute of Phytomedicine, University of Hohenheim, Stuttgart, Germany; ^7^Department of Phytopathology, Institute of Phytomedicine, University of Hohenheim, Stuttgart, Germany; ^8^Department of Food Chemistry and Analytical Chemistry, Institute of Food Chemistry, University of Hohenheim, Stuttgart, Germany; ^9^Department of Biology, Section of Microbiology, University of Copenhagen, Copenhagen, Denmark

**Keywords:** fertilization management, root exudates, rhizosphere microbiota, high-throughout amplicon sequencing, 16S rRNA, fungal ITS2 region, stress-related gene expression

## Abstract

Fertilization management can affect plant performance and soil microbiota, involving still poorly understood rhizosphere interactions. We hypothesized that fertilization practice exerts specific effects on rhizodeposition with consequences for recruitment of rhizosphere microbiota and plant performance. To address this hypothesis, we conducted a minirhizotron experiment using lettuce as model plant and field soils with contrasting properties from two long-term field experiments (HUB-LTE: loamy sand, DOK-LTE: silty loam) with organic and mineral fertilization history. Increased relative abundance of plant-beneficial arbuscular mycorrhizal fungi and fungal pathotrophs were characteristic of the rhizospheres in the organically managed soils (HU-org; BIODYN2). Accordingly, defense-related genes were systemically expressed in shoot tissues of the respective plants. As a site-specific effect, high relative occurrence of the fungal lettuce pathogen *Olpidium* sp. (76–90%) was recorded in the rhizosphere, both under long-term organic and mineral fertilization at the DOK-LTE site, likely supporting *Olpidium* infection due to a lower water drainage potential compared to the sandy HUB-LTE soils. However, plant growth depressions and *Olpidium* infection were exclusively recorded in the BIODYN2 soil with organic fertilization history. This was associated with a drastic (87–97%) reduction in rhizosphere abundance of potentially plant-beneficial microbiota (*Pseudomonadaceae*, *Mortierella elongata*) and reduced concentrations of the antifungal root exudate benzoate, known to be increased in presence of *Pseudomonas* spp. In contrast, high relative abundance of *Pseudomonadaceae* (Gammaproteobacteria) in the rhizosphere of plants grown in soils with long-term mineral fertilization (61–74%) coincided with high rhizosphere concentrations of chemotactic dicarboxylates (succinate, malate) and a high C (sugar)/N (amino acid) ratio, known to support the growth of Gammaproteobacteria. This was related with generally lower systemic expression of plant defense genes as compared with organic fertilization history. Our results suggest a complex network of belowground interactions among root exudates, site-specific factors and rhizosphere microbiota, modulating the impact of fertilization management with consequences for plant health and performance.

## Introduction

Fertilization practices are a central component of agricultural management having a strong impact on plant performance including crop yield and quality, plant health and resistance against abiotic and biotic stresses. However, limitations in the adaptation of fertilizer inputs to actual crop demands can cause undesirable environmental effects, such as nutrient leaching, alteration of soil pH, eutrophication of surface waters, emission of greenhouse gases, and soil degradation (Loreau et al., 2001; Foley et al., 2005; Robertson and Vitousek, 2009; Geisseler and Scow, 2014). Due to the rising demand for more sustainable agricultural production systems with reduced inputs of agrochemicals and the closing of nutrient cycles to counteract detrimental ecological side effects, investigations into alternative management practices gain in importance. The availability of essential plant nutrients in farmland is controlled by interactions between fertilization management and microbial processes (Schmidt et al., 2019). Nearly all relevant soil processes are influenced by microbial activities (Mäder et al., 2002), which underlines the importance of considering plant–microbial interactions in this context. Various beneficial aspects have been linked with organic fertilization strategies, such as increased soil organic matter, stimulation of microbial activity (Lori et al., 2017), increased microbial biomass and diversity (Esperschütz et al., 2007; Hartmann et al., 2014; Francioli et al., 2016; Schmid et al., 2018), and enrichment in plant–beneficial microorganisms (Francioli et al., 2016). However, these findings cannot be generalized and are influenced by additional factors, such as soil properties, climatic conditions or specific practices of crop management. In addition, plant roots are similarly powerful drivers of the assemblage of the rhizosphere microbial community, which exhibits distinct structural and functional differences compared with the bulk soil microbiota (Turner et al., 2013). Organic rhizodeposition shapes rhizosphere microbiota (Walters et al., 2003; Narula et al., 2009) by providing nutrients, signaling compounds, and bio-active substances against pests and pathogens (Doornbos et al., 2012; Chaparro et al., 2013; Baetz and Martinoia, 2014; Windisch et al., 2017). The quantity and composition of rhizodeposits are highly variable and influenced by soil texture, plant nutritional status, abiotic and biotic stress factors, plant genotype, and rhizosphere microbiota (Neumann and Römheld, 2007). However, the impact of fertilization management on rhizodeposits, triggering the selective recruitment of rhizosphere microbiota with potential feed-back loops on plant performance and health status, still remains an open question.

A recent study demonstrated that the fertilization legacy of the soil contributed to the assemblage of rhizosphere bacterial communities in lettuce (Chowdhury et al., 2019). The crucial role of the rhizosphere microbiota for plant performance and health has been comprehensively reviewed (Berendsen et al., 2012; Berg et al., 2016). Furthermore, a relationship between plant health and agricultural management conferred via soil microorganisms has been postulated (Lapsansky et al., 2016; van der Putten et al., 2016; Bakker et al., 2018). Chowdhury et al. (2019) provided first experimental evidence for induction of physiological adaptations under long-term organic vs. mineral fertilization that helped lettuce plants to cope with environmental stresses. A better understanding of these interactions, triggering plant–beneficial interactions but also detrimental rhizosphere effects, could therefore contribute to the development of practical approaches toward improved crop productivity and agroecosystem sustainability (Schmid et al., 2018) according to the concept of “soil biological engineering” (Bender et al., 2016). However, a more detailed understanding particularly of the critical plant factors determining these interactions still represents a major knowledge gap.

In this context, our study was initiated as a complementary approach to the recently published study by Chowdhury et al. (2019). Soils with contrasting physicochemical properties and long-term organic or mineral fertilization histories were investigated. Bacterial, archaeal and fungal communities, the composition of organic compounds in the rhizosphere soil solution as well as plant performance and expression of stress-related genes were analyzed to obtain a more holistic picture of plant-microbe interactions.

We hypothesized that long-term fertilization practices will result in characteristic patterns and chemical composition of the rhizosphere soil solution, with impact on soil microbiota and the recruitment of rhizosphere microbiota. This would affect the performance and health of the model plant lettuce. The final aim was the identification of rhizosphere metabolite profiles characteristic of the investigated long-term fertilization strategies potentially related to alterations in the assemblage of rhizosphere microbiota and plant performance.

## Materials and Methods

### Soil Sampling and Setup of the Minirhizotron Experiment

Field soils with contrasting properties in terms of soil type and fertilization history, originating from the two long-term fertilization experiments DOK-LTE belonging to the Research Institute of Organic Agriculture (FiBL; Therwil, Switzerland since 1978) and HUB-LTE belonging to the Humboldt-Universität zu Berlin (HUB; Thyrow, Germany since 2006) were used for plant cultivation. The field trial (i) DOK-LTE on a Haplic Luvisol (silty loam) compares bio-dynamic (compost and manure fertilizers with biodynamic preparations; BIODYN2) vs. full mineral NPK (CONMIN) fertilization. The fertilization intensity of the organic system in BIODYN2 was based on the fodder produced in the crop rotation and reflects the intensity of Swiss organic farms. The mineral fertilizer level in CONMIN was adjusted to plant-specific Swiss standard recommendations (Mäder et al., 2002). The field trial (ii) HUB-LTE on a Retisol (loamy sand) compares application of organic farmyard manure (green, and cattle manure; HU-org) vs. full mineral NPK (HU-min) fertilization. The mineral fertilizer level in HU-min was adjusted to soil fertility and yield performance in the northeast of Germany. Nitrogen was applied as calcium ammonium nitrate (KAS), phosphorus as triple-superphosphate (TSP) and potassium as Patentkali^®^ (K + S Minerals and Agriculture GmbH, Kassel, Germany) besides an incorporation of harvested straw of winter and cover crops. The fertilization intensity of the organic system in HU-org was based on cattle and green manure from cover crops with legumes mixture. In order to obtain field soil for the minirhizotron experiment, soil sampling was performed after harvest of standing crops from the respective upper 30 cm soil layer from each field trial and combined as representative sample of 15 sampling spots. For homogenization, the soil was air-dried, sieved (4 mm mesh size) and stored in the dark at 7°C. For reactivation of the microbial communities prior to the experiment, experimental soil and soil as control without lettuce cultivation (bulk soil) was incubated for two weeks in the dark with a 20°C day/15°C night temperature regime at 100-hPA water potential (T5 tensiometer, UMS, AG, München, Germany). Detailed soil characteristics, management practices, and physicochemical parameters of the experimental soils are summarized in Table 1.

**TABLE 1:** Characteristics of the field soils **(A)** and physicochemical parameters of bulk soils **(B)**.

	**HUB-LTE**		**DOK-LTE**	
	**HU-org**	**HU-min**	**BIODYN2**	**CONMIN**
**A. Experimental soil and cultivation characteristics**^1,2^				
Fertilization management	Composted farmyard manure, K as Patentkali, liming as CaCO_3_-MgCO_3_	Standard practice of mineral fertilizer (NPK)^5^, liming as CaCO_3_-MgCO_3_	Composted farmyard manure (1.4 livestock unit (LU) ha^–1^ year^–1^), biodynamic preparations^3^	Standard practice of mineral fertilizer (NPK)^4^

**Soil management**	**Tillage**	**Tillage**	**Tillage**	**Tillage**

Crop protection	No pesticides, mechanical weed control	Mechanical weed control with chemical pesticides application	Mechanical weed control, plant extracts and biodynamic preparations, biocontrol (*Bacillus thuringiensis* subsp.)	Mechanical weed control with herbicide and chemical pesticides application

**Soil type**	**Retisol (loamy sand)**		**Haplic Luvisol (silty loam)**	

**Soil texture (0-30 cm)**				
Clay (<2 μm) [%]	3		15	
Silt (2–63 μm) (%)	14		70	
Sand (63–2,000 μm) (%)	83		15	

*The soils were obtained in 2016 from two long-term field experiments (LTEs) located at different field sites (DOK-LTE in Therwil, Switzerland; HUB-LTE in Thyrow, Germany) and incubated together with planted soils under the same growth chamber conditions. Data represent means ± standard errors of four independent replicates. Different lowercase letters indicate significant differences between organic vs. mineral fertilization tested separately for the sites DOK-LTE and HUB-LTE by *t*-test, *p* ≤ 0.05. ^1^Department of Soil Sciences, Research Institute of Organic Agriculture (FiBL), Frick, Switzerland; ^2^Experiment Thy_ABS “Cropping Systems” in Thyrow, Albrecht Daniel Thaer-Institute of Humboldt-Universität zu Berlin; ^3^Hartmann et al., 2014; ^4^Mäder et al., 2002, quantities for NPK fertilizer in CONMIN: Nitrogen soluble (kg N ha^–1^ year^–1^) 125, Phosphorus (kg P ha^–1^ year^–1^) 42, Potassium (kg K ha^–1^ year^–1^) 253. ^5^Quantities for NPK fertilizer in HU-min: Nitrogen soluble (kg N ha^–1^ year^–1^) 128, Phosphorus (kg P ha^–1^ year^–1^) 21, Potassium (kg K ha^–1^ year^–1^) 128.*				

	**HUB-LTE**		**DOK-LTE**	
	**HU-org**	**HU-min**	**BIODYN2**	**CONMIN**

**B. Physicochemical parameters – Bulk soil**				
pH (CaCl_2_)	6.50 ± 0.12 a	6.54 ± 0.04 a	6.68 ± 0.02 a	6.30 ± 0.03 b
Corg (%)	0.81 ± 0.01 a	0.77 ± 0.01 a	1.72 ± 0.01 a	1.42 ± 0.01 b
Cmic (μg g^–1^)	103.23 ± 7.83 a	95.48 ± 10.45 a	446.97 ± 22.17 a	327.86 ± 1.53 a
C/N	9.16 ± 0.56 a	9.63 ± 0.65 a	8.06 ± 0.19 a	7.64 ± 0.23 a
Electrical conductivity (EC) (μS cm^–1^)	357.60 ± 29.01 a	356.87 ± 46.94 a	442.00 ± 40.57 a	443.90 ± 51.63 a

(mg kg^–1^ soil)				
C total	8197.89 ± 18.41 a	7793.92 ± 7.59 a	17836.47 ± 4.41 a	14563.65 ± 4.34 b
N total	906.94 ± 5.44 a	821.70 ± 4.70 a	2217.17 ± 5.09 a	1912.11 ± 5.54 b
NO_3–_-N	362.01 ± 8.55 a	249.66 ± 6.60 a	442.55 ± 14.46 a	321.75 ± 8.91 a
DL P	92.90 ± 0.09 b	102.95 ± 0.17 a	26.32 ± 0.03 b	37.10 ± 0.03 a
DL K	75.778 ± 0.25 b	124.80 ± 0.96 a	96.30 ± 0.38 a	81.62 ± 0.29 b
Mg	71.33 ± 0.68 a	72.59 ± 0.23 a	153.34 ± 0.61 b	218.87 ± 0.54 a
Na	53.54 ± 1.27 a	32.72 ± 0.32 a	45.78 ± 0.28 b	136.00 ± 0.47 a
Cu	1.99 ± 0.00 a	1.81 ± 0.00 b	4.96 ± 0.01 b	5.81 ± 0.00 a
Fe	189.20 ± 0.25 a	190.80 ± 0.22 a	201.80 ± 0.21 b	214.00 ± 0.26 a
Mn	51.02 ± 0.12 a	49.60 ± 0.09 a	253.60 ± 0.63 a	240.40 ± 0.83 a
Zn	5.59 ± 0.01 a	5.54 ± 0.01 a	5.87 ± 0.01 a	3.45 ± 0.00 b

*Corg, organic carbon; Cmic, microbial carbon; DL P and DL K, double-lactate extraction for P and K, (VDLUFA, 2020).*				

Lettuce (*Lactuca sativa* L. cv. Tizian, Syngenta, Bad Salzuflen, Germany) cultivation, harvest, and root exudate sampling were performed as described by Neumann et al. (2014). To achieve homogenous plant development, lettuce seedlings were pre-cultivated until the five-leaf stage (BBCH 15) in a soil-sand mixture (70/30: w/w). Thereafter, the seedlings were transferred to minirhizotrons (0.6 kg of the soil–sand mixture 70/30) made from PVC tubes (height 22 cm; diameter 9 cm) with transparent root observation windows. The minirhizotrons were fixed at an angle of 45° to stimulate root growth along the root observation window for exudate sampling.

Full mineral N fertilization (517 mg N kg^–1^ substrate as YaraLiva Calcinit [Ca(NO_3_)_2_], Yara, Oslo, Norway) was supplied to cover the plant demand during the culture period. The first half of the recommended N amount for lettuce was supplied after seedling transfer to minirhizotrons, the second half provided two weeks later. The soil moisture level was adjusted to 18–20% w/w by addition of demineralized water (25 ml kg^–1^ soil) every second day throughout the culture period. Lettuce seedlings were cultivated in a growth chamber with a 16 h light period at 420 μmol m^–2^ s^–1^, 60% relative humidity, and a 20°C/15°C day/night temperature regime, with eight replicates per treatment in a randomized block design. Biomass of plants, root characteristics and nutritional status of lettuce shoots were analyzed. To confirm general treatment responses in plant growth and visual symptoms of pathogen infections, the experiment was performed twice with cultivation periods of six and nine weeks, respectively, with a more detailed analysis of soil microbiota, rhizosphere chemistry and gene expression after nine weeks.

### Analysis of Mineral Nutrients in Soil and Shoot Tissue

The content of macro- and micronutrients in bulk soil and plant samples were analyzed according to the certified protocols of the Association of German Agricultural Analytic and Research Institutes, VDLUFA, Germany (VDLUFA, 2020). After determination of fresh shoot biomass, shoots and soil samples were oven-dried at 60°C for three days and dry biomass was recorded. Subsequently, shoots were stored in a desiccator for another two days and 200–500 mg of dry plant material and 4 g of soil were subjected to microwave digestion (Mars 6, CEM, Charlotte, NC, United States) with 5 ml of HNO_3_ (65%) and 3 ml of H_2_O_2_ (30%) for 20–30 min.

Potasium, Na, P, Mg, Fe, Mn, Cu, and Zn concentrations in shoot tissue and soils were determined via inductively coupled plasma optical emission spectrometry (ICP-OES); total C and N were determined via elemental analysis (Elementary Vario El cube, Elementar, Langenselbold, Germany). Soil pH was determined in calcium chloride solution and salinity via electrical conductivity.

### Root Morphology

For analysis of root morphological characteristics, roots of four replicates were washed from soil using sieves (mesh size 0.5–1.0 mm) and fresh and dry biomass were recorded. Morphological characteristics were determined from fresh root samples, stored in 60% (v/v) ethanol. For analysis, root samples submerged in a water film on transparent Perspex trays, were separated with forceps and subsequently digitized using a flat-bed scanner (Epson Expression 1000 XL, Tokyo, Japan). Root length, average root diameter and the proportion of fine roots of the digitized samples were measured by applying the WinRHIZO root analysis software (Regent Instruments, Quebec, QC, Canada). Root hair length was recorded non-destructively along the root observation plane of the minirhizotrons by a video microscope (Stemi 200-c, Zeiss, Oberkochen, Germany). The digitized video images were analyzed using the AxioVision, software, Version 3.1.2.1 (Zeiss, Oberkochen, Germany).

### Plant Gene Expression

For gene expression studies, leaf samples were obtained from plants in quadruplicates. Four representative leaves per plant were pooled and snap-frozen in liquid nitrogen (Chowdhury et al., 2019). Homogenized leaf material (100 mg) was subjected to total RNA extraction using the RNeasy Plant Mini Kit (QIAGEN GmbH, Hilden, Germany). RNA was quantified by a NanoDrop spectrophotometer (Thermo Fisher Scientific, Waltham, MA, United States). Target genes of lettuce (Chowdhury et al., 2019) were selected based on comparisons with functional genes from *Arabidopsis thaliana* using “The Arabidopsis Information Resource” (Berardini et al., 2015)^1^. The reference gene glyceraldehyde-3-dehydrogenase was used for normalization of qPCR results. The primer pairs for qPCR were designed using the Primer3Plus software (Untergasser et al., 2007). cDNA was synthesized from 2 μg of total RNA with the High Capacity cDNA Reverse Transcription Kit with RNase Inhibitor (Applied Biosystems, Foster City, CA, United States). The qPCR was performed with Power SYBR Green Supermix (Applied Biosystems, Foster City, CA, United States) using a peqSTAR 96Q thermal cycler (PEQLAB Biotechnologie, Erlangen, Germany). cDNA dilutions (1 μl, 1:4) were used as PCR templates. Each PCR reaction contained 12.5 μl of 2 × Power SYBR Green Supermix, 0.4 μM primers (Eurofins MVG Operon, Ebersberg, Germany), and 1 μl of template in a 25-μl reaction. PCR reactions were heated to 95°C for 3 min and then for 40 cycles with steps of 95°C for 30 s and 60°C for 30 s. The generation of specific PCR products was confirmed by melting curve analysis and gel electrophoresis. The genes, their primer pairs and qPCR conditions used in this study are described in Supplementary Table 1. The 2–ΔΔCt method (Livak and Schmittgen, 2001) was employed for relative quantification. Normalization to the endogenous control for each condition was followed by logarithmic transformation to fold change differences. The standard error of the mean was calculated from the average of technical triplicates, obtained from each of four biological replicates (*n* = 4).

### Sampling of Rhizosphere Soil Solutions

Samples of the rhizosphere soil solution were collected with moist sorption filters (5 mm Ø, filter paper: MN815, Macherey-Nagel, Düren, Germany) placed onto the surface of lateral roots growing along the root observation window according to Haase et al. (2007). Micro-sampling was conducted nine weeks after sowing during vegetative growth of the lettuce plants, to account for most active carbohydrate partitioning to the roots and high root exudation during this phase (Marschner, 1995). For each minirhizotron, rhizosphere sampling was conducted with two 5 mm sorption filters (equivalent to 1 cm root length), applied in triplicate in subapical root zones (1–2 cm behind the root tip) and basal root zones (older, mature parts of the root system, 8–9 cm behind the root tip). As a control, sampling was performed in soil zones without visible root development (soil without root contact). Samples for each minirhizotron were pooled after an incubation time of 4 h and kept frozen at −20°C (Neumann et al., 2014). Rhizosphere soil solution was extracted from sorption filters with 0.6 ml of acetonitrile: H_2_O (1:1).

#### Analysis of Carboxylates

Aliquots of 40 μl obtained from 0.6 ml sorption filter extract were evaporated to dryness at 30°C, using a SpeedVac Concentrator (Savant, Farmington, CT, United States) and re-dissolved in 400 μl of high performance liquid chromatography (HPLC) elution buffer (18 mM KH_2_PO_4_, pH 2.1 adjusted with H_3_PO_4_; Neumann, 2006). Carboxylates were determined according to the method described by Neumann (2006), using RP-HPLC analysis in the ion suppression mode with isocratic elution (18 mM KH_2_PO_4_, pH 2.1). The identification and quantitative determination as organic acids (acetic, malic, lactic, citric, succinic, and fumaric acid) were conducted, using a reversed phase C-18 column (GROM-SIL 120 ODS ST, 5 μm particle size, 290 mm × 4.6 mm equipped with a 20 mm × 4.6 mm guard column with the same stationary phase, Grom, Herrenberg, Germany) with direct UV detection at 210 nm and comparison with known standards. In representative samples, the identity of detected carboxylates was additionally confirmed by commercial enzymatic tests (r-Biopharm, Darmstadt, Germany).

#### Analysis of Sugars

Aliquots of 400 μl from 0.6 ml sorption filter extract were evaporated to dryness at 55°C, using nitrogen evaporation and re-dissolved in 40 μl acetonitrile: H_2_O (70:30). Analyses of sugars (fructose, glucose, maltose, and sucrose) in rhizosphere soil solutions of lettuce were performed by HPLC-Evaporative Light Scattering Detector (ELSD) with isocratic elution (acetonitrile: H_2_O, 75:25) on a Perkin Elmer Series 200 HPLC system with a Sedex Model 80 LT ELSD system (Sedere, Orléans, France) equipped with a Shodex, Ashipak NH2P-40 3E column, 5 μm particle size, 250 mm × 3.0 mm (Shodex, München, Germany) and external standards.

#### Analysis of Amino Acids

A total of 20 μl aliquots from 0.6 ml sorption filter extract were mixed with 15 μl of derivatization agent (ACCQFLUOR REAG, Waters, Milford, MA, United States) and 65 μl of borate buffer, incubated 10 min at 55°C, followed by addition of 400 μl acetonitrile: H_2_O (1:4) with modifications of the method of Cohen and Michaud (1993). Determination of amino acids (glutamic acid, asparagine, serine, glutamine, glycine, threonine, histidine, alanine, proline, cysteine, thyrosine, methionine, isoleucine, leucine, and phenylalanine) was performed by HPLC-MS, using a Velos LTQSystem (Thermo Fisher Scientific, Waltham, MA, United States) equipped with a ACCUTAG^TM^ column, 4 μm particle size, 150 mm × 3.9 mm (Waters, Milford, MA, United States) and additional comparison with external standards. Gradient elution was performed with (A) ammonium formate: methanol: H_2_O (40:9:60) and (B) acetonitrile.

#### Analysis of Benzoate

For determination of the antifungal compound benzoate 50 μl aliquots of sorption filter extract were mixed with 50 μl of H_2_O followed by UHPLC-MS analysis on a Velos LTQSystem (Thermo Fisher Scientific, Waltham, MA, United States). Identification and quantitative analysis were conducted on an ACCLAIM^TM^C30 column, 150 mm × 3 mm (ThermoScientific, Waltham, MA, United States) with gradient elution (solvent A): H_2_O: acetonitrile (95:5) and (solvent B): acetonitrile and an external standard.

### Microbial Community Analyses

#### Total Community – DNA Extraction

The roots of the respective plants, which were studied for gene expression, were used for analysis of rhizosphere microbiota (bacteria, archaea, and fungi). At first, roots were washed with sterile tap water (Schreiter et al., 2014b). The rhizosphere fraction was obtained from 5 g of representative root samples, which were collected from complete root systems, by 1 min Stomacher treatment (Seward Ltd., Worthing, United Kingdom) followed by centrifugation (Schreiter et al., 2014a). Rhizosphere pellets were kept at −20°C for total community (TC)-DNA extraction. Root-associated soil was sampled from the soil fraction loosely adhering to the root system collected after vigorous shaking. After collection, the soil samples were immediately frozen at −20°C.

Total community-DNA was extracted from root-associated soil (0.5 g) and rhizosphere pellets using the FastPrep-24 bead-beating system and FastDNA Spin Kit for Soil. DNAs were purified with the GeneClean Spin Kit (both MP Biomedicals, Santa Ana, CA, United States).

#### Bacterial and Archaeal Communities

Bacterial and archaeal communities in root-associated soil and rhizosphere were characterized based on sequencing of the V3-V4 region of 16S rRNA genes amplified using the primer pair 341F (5′-CCTAYGGGRBGCASCAG-3′) and 806R (5′-GGACTACHVGGGTWTCTAAT-3′) originally published by Yu et al. (2005) and modified by Sundberg et al. (2013) or Caporaso et al. (2011), respectively, targeting both Bacteria and Archaea kingdoms. Detailed description of PCR amplification, purification, normalization, and amplicon sequencing on an Illumina^®^ MiSeq^®^ platform (2 × 250 cycles; Illumina Inc., San Diego, CA, United States) can be found in the Supplementary Text 1.

Unassembled raw amplicon data were submitted to NCBI Sequence Read Archive (SRA)^2^ under accession number PRJNA622892. Raw sequence reads were first trimmed of primer sequences used in first PCR using cutadapt (Martin, 2011) and only read pairs for which both primers were found were retained for subsequent analysis. Primer trimmed sequences were then merged, clustered in operational taxonomic units (OTUs) using UPARSE-OTU algorithm (Edgar, 2013) and a 97% pairwise sequence similarity threshold. The taxonomic annotation of each cluster representative sequence was performed using mothur classify.seq function (Schloss et al., 2009) with default parameters and the Ribosomal Database Project trainset 14 formatted for mothur (Cole et al., 2014)^3^. Only annotations with a confidence threshold above 80% were considered. Sequences classified as chloroplasts, mitochondria, or unclassified at the domain level were removed, resulting in a total of 7,294 OTUs. The average number of quality-filtered sequences per sample was 19,969.

#### Fungal Communities

High-throughput sequencing based on the Internal Transcribed Spacer (ITS2) region was conducted in root-associated soil and rhizosphere. PCRs using the sample-specific barcoded NGS-primer-pair ITS86F (5′-GTGAATCATCGAATCTTTGAA-3′; Op De Beeck et al., 2014) and ITS4 (5′-TCCTCCGCTTATTGATATGC-3′; White et al., 1990) as well as the processing of the ITS2 amplicon pool on an Illumina^®^ MiSeq^®^ platform (paired-end mode, 2 × 300bp) were carried out as previously described with a few modifications (Sommermann et al., 2018). Detailed information of PCR amplification and sequencing can be found in the Supplementary Text 2.

Unassembled raw amplicon reads were submitted to European Nucleotide Archive (ENA)^4^ under BioProject accession number PRJEB39853. Barcode, primer and adapter trimming were performed based on a customized in-house perl script including the FASTX toolkit^5^ followed by raw sequence merging using FLASH (Magoč and Salzberg, 2011). Subsequently, the analysis of the resulting sequences was carried out with a local version of the GALAXY Bioinformatics Platform^6^ based on a database-dependent strategy (Antweiler et al., 2017) using UNITE database v7.2 (UNITE Community, 2017) by applying the closed reference approach (Carter et al., 2017). All sequences were aligned with the database (e-value 0.001) and only results with an alignment length >200 bp and a similarity >97% to the reference were kept. Furthermore, BLAST-PARSER (Antweiler et al., 2017) was used for taxonomic assignment based on the lowest e-value. The fungal OTU abundance table was generated by counting the sequences per assignment and using the SH-numbers from the database as identifier. Sequences not classified to the kingdom “fungi” (0.17%) were removed from the fungal OTU-table. Finally, a total of 1,159 OTUs was obtained with an average of 167,113 high quality sequence reads per sample.

### Detection of Pathogen Infection in the Root Tissue

Colonization of *Olpidium* sp. in lettuce roots was assessed according to the method described by De Cara et al. (2008) with modifications. Soil adhering to seedling roots was removed by washing with running water for 5 min. Subsequently, the roots were assessed visually for root discoloration and small sections (2-4 mm) were randomly excised from the roots of each replicate. Root specimens were then transferred to microscopic slides, mounted with lactophenol aniline blue solution (Merck, Darmstadt, Germany) and examined for the presence of sporangia and resting spores in epidermal cells using an Axioskop microscope (Carl Zeiss Microscopy GmbH, Jena, Germany) equipped with an Axiocam camera and AxioVision SE64 Rel. 4.8 software.

### Statistical Analysis

For the statistical analysis of significant differences of the nutritional status of lettuce between treatment groups (separately for each LTE), a one-way ANOVA (factor fertilization) followed by a Tukey-test (*p* ≤ 0.05 significance level) was performed using the SAS software 9.4 (Institute Inc., Cary, NC, United States). For the statistical evaluation of physicochemical parameters (bulk soil), the expression of stress-related genes in lettuce leaves, and the chemical composition of rhizosphere soil solution, *t*-test and Tukey’s HSD pairwise testing was applied. Data are presented as means ± standard errors (SE).

Multivariate analyses of microbial communities were carried out by R using the packages *edgeR* (Robinson et al., 2010; McCarthy et al., 2012), *vegan* (Oksanen et al., 2019), *MASS* (Venables and Ripley, 2002), *ggplot2* (Wickham, 2016), *phyloseq* (McMurdie and Holmes, 2013), *pheatmap* (Kolde, 2019), *gplots* (Warnes et al., 2019), *car* (Fox and Weisberg, 2019), and *agricolae* (De Mendiburu, 2020). Alpha-diversity indices (species richness, Shannon, Pielou) were averaged per replicate over 100 randomly taken subsamples of a size corresponding to the sample with the lowest number of reads in the complete dataset (=6,744 for 16S rRNA gene, 96,750 for ITS). Indices were tested for the effect of microhabitat (root-associated soil and rhizosphere) or fertilization management, respectively, by pairwise *t*-test (*p* ≤ 0.05). Non-metric multidimensional scaling (NMDS) was used to ordinate similarity between microbial communities based on relative abundances (Bray–Curtis distance). The effect of site, fertilization and habitat on the microbial community composition was tested by PERMANOVA analysis based on relative abundances (10,000 permutations, Bray–Curtis distance). The non-rarefied community data were checked for differentially abundant microbial taxa between different fertilization managements. Data were analyzed by likelihood ratio tests under negative binomial distribution and generalized linear models (edgeR). In doing so, only taxa with presence in at least three samples over the total dataset using a FDR-corrected *p* ≤ 0.05 were considered. In order to study the potential relationship between rhizosphere soil solution and the bacterial and archaeal as well as fungal community composition, canonical correspondence analyses (CCA, 999 permutations) were carried out on log10 transformed relative abundances. After checking for linear dependency, environmental variables (organic compounds averaged over basal and subapical root) were fitted onto the CCA ordination by the envfit function (999 permutations). In addition, log transformed relative abundances of *Pseudomonadaceae* OTUs and fungal OTUs at least classified at genus level (both relative abundance >0.5%) that were significantly enriched (FDR < 0.05) in minerally fertilized soils (tested separately per site; edgeR) were included in envfit. The relative abundance of prevalent genera was graphically displayed in a heatmap (clustering of rows using Euclidean distance). The FUNGuild database (Nguyen et al., 2016) was used to categorize fungal communities at genus level into trophic modes (saprotroph, symbiotroph and pathotroph) and into different guilds for further classification.

Biplots of principle component analysis (PCA) were used to ordinate composition of chemical compounds of the rhizosphere soil solutions, related to their habitat and tested for the effect of fertilization management. The distribution of the data was graphically displayed in a p-dimensional cartesian coordinate system with Euclidean distance by using R Studio software 3.4.1 and prcomp and autoplot functions.

## Results

### Plant Biomass, Root Growth Characteristics, and Nutritional Status

**FIGURE 1:**
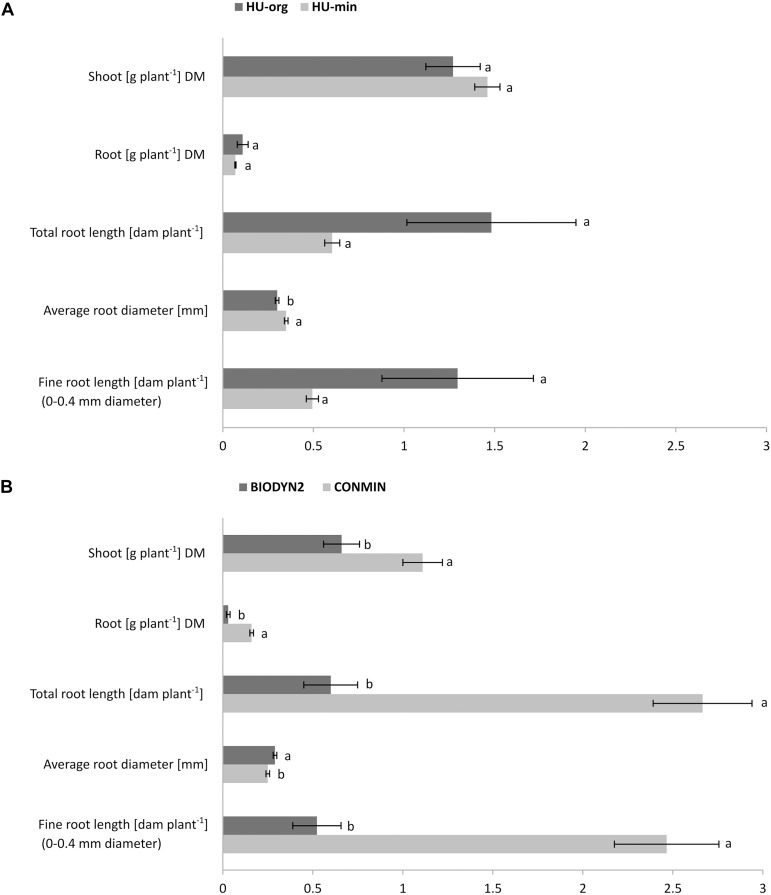
Shoot and root dry biomass (DM) and root morphology of lettuce (cv. Tizian). The plants were grown in minirhizotron culture for nine weeks in soils with long-term organic (HU-org, BIODYN2) or mineral (HU-min, CONMIN) fertilization history. Means ± standard errors of four independent replicates. Different lowercase letters indicate significant differences between organic vs. mineral fertilization tested separately for the sites HUB-LTE **(A)** and DOK-LTE **(B)** by one-way ANOVA, Tukey’s HSD pairwise test, *p* ≤ 0.05.

There were no significant differences for shoot and root dry biomasses of lettuce grown in HU-org vs. HU-min soil (Figure 1A). However, a significant decline in shoot and root dry biomass (by 41% and 81%) of lettuce grown in the BIODYN2 soil with organic fertilization was recorded compared to the CONMIN soil supplied with mineral fertilizers (Figure 1B). Growth depression was confirmed in two independent experiments in the BIODYN2 soil (Supplementary Table 2) and corresponded with a significant decline in total root length and fine root length (<0.4 mm diameter) (Figure 1B and Supplementary Table 2).

At the end of the culture period, shoot nutrient concentrations were recorded and deficiencies in nutrient elements such as P and K were identified in all treatments. For K, significant differences related to the fertilization management were observed, showing higher values in the HU-min vs. HU-org soil and higher levels in BIODYN2 compared to CONMIN soil. Other macro- and micronutrients in the shoot tissues, such as Ca, Mg, Mn, and Cu reached the sufficiency range in all treatments. A very moderate limitation was detected for N in all treatments. Iron concentrations were generally high, with particularly high values in DOK-LTE soils (401–842 mg kg^–1^ dry biomass, Table 2). Significant differences in the nutritional status were more pronounced for lettuce grown in DOK-LTE compared to HUB-LTE soils.

**TABLE 2:** Plant nutritional status of lettuce (cv. Tizian).

**Nutrient concentration of shoot dry biomass (DM) of lettuce (cv. Tizian)**					
		**HUB-LTE**		**DOK-LTE**	
		**HU-org**	**HU-min**	**BIODYN2**	**CONMIN**

**Macronutrients (g kg^–1^ shoot DM)**					
N	35*	31.40 ± 1.51 a	31.51 ± 1.60 a	30.68 ± 0.40 a	32.04 ± 1.21 a
P	3.0*	1.66 ± 0.25 a	2.06 ± 0.09 a	1.75 ± 0.03 a	1.91 ± 0.12 a
K	42*	20.23 ± 3.18 b	33.44 ± 0.58 a	38.11 ± 1.93 a	26.35 ± 1.47 b
Ca	12*	17.46 ± 3.11 a	18.42 ± 1.60 a	14.76 ± 0.45 b	16.59 ± 0.62 a
Mg	1.0*	3.79 ± 0.61 a	3.54 ± 0.21 a	3.53 ± 0.10 b	5.05 ± 0.29 a
S	2.5*	1.79 ± 0.27 a	2.06 ± 0.13 a	2.54 ± 0.08 a	2.36 ± 0.12 a
Na	0.6*	4.08 ± 0.93 a	2.92 ± 0.05 a	3.40 ± 0.35 b	5.59 ± 0.25 a
**Micronutrients (mg kg^–1^ shoot DM)**					
Cu	2.5**	2.87 ± 0.55 a	3.21 ± 0.41 a	7.10 ± 0.53 a	7.52 ± 0.64 a
Fe	50**	123.63 ± 18.00 a	289.38 ± 122.81 a	841.52 ± 203.32 a	401.24 ± 162.02 a
Mn	20**	63.37 ± 9.78 a	85.91 ± 9.48 a	80.10 ± 5.74 a	81.75 ± 12.19 a
Zn	20**	19.74 ± 2.96 a	26.47 ± 2.00 a	55.37 ± 2.06 a	33.55 ± 3.91 b

The plants were grown in minirhizotron culture for nine weeks in soils with long-term organic (HU-org, BIODYN2) or mineral (HU-min, CONMIN) fertilization history. Data represent means ± standard errors of four independent replicates per treatment. Different lowercase letters indicate significant differences between organic vs. mineral fertilization tested separately for the sites DOK-LTE and HUB-LTE by one-way ANOVA, Tukey’s HSD pairwise test, *p* ≤ 0.05. *Deficiency threshold macronutrients (g kg^–1^ DM). **Deficiency threshold micronutrients (mg kg^–1^ DM), (Bergmann, 1988).

### Expression of Stress-Related Genes in the Shoot Tissue

**FIGURE 2:**
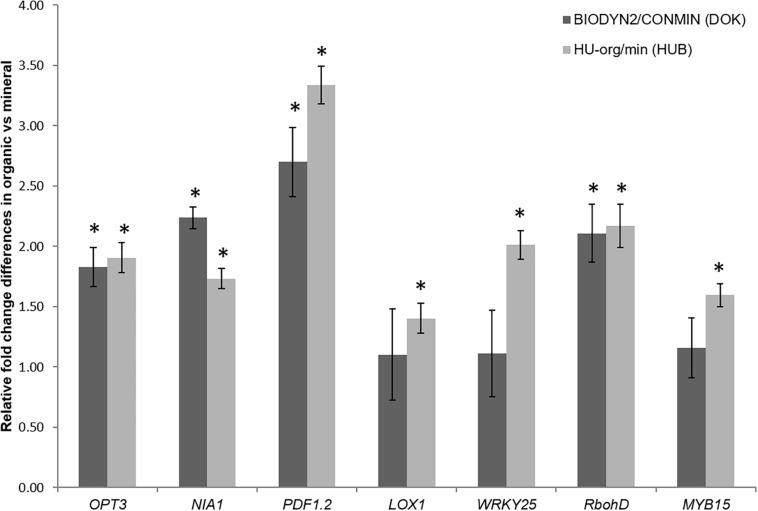
Upregulated genes in shoots of lettuce grown over a period of nine weeks in soils with long-term organic (HU-org, BIODYN) in comparison to mineral (HU-min, CONMIN) fertilization history. The 2^–ΔΔ*Ct*^ method (Livak and Schmittgen, 2001) was employed for relative quantification (*n* = 4) by qPCR (see section “Materials and Methods”). Means ± standard errors of four independent replicates showing relative changes in gene expression of plants grown under long-term organic vs. mineral fertilization. Only genes showing significant (*p* ≤ 0.05) differences in ΔCt values between organic vs. mineral fertilization within each site as revealed by Tukey’s HSD pairwise test are shown (denoted by asterisks). Gene names, putative functions and primer sequences are described in Supplementary Table 1.

A qPCR-based method was used to investigate the relative expression of 14 genes, known to be involved in biotic or abiotic stress signaling pathways as previously investigated for lettuce (Chowdhury et al., 2019). Although various genes showed similar expression in plants grown in both soils with long-term organic fertilization (HU-org, BIODYN2) and long-term mineral fertilization (HU-min, CONMIN), certain stress-related genes were significantly upregulated in the treatments with long-term organic fertilization at both field sites. The results showed a significantly enhanced expression of the genes *RbohD*, *PDF1.2*, the Fe-transporter *OPT3* gene and the nitrate reductase gene *NIA1* in shoots of lettuce grown in soils with long-term organic fertilization (HU-org, BIODYN2) in comparison to plants grown in soils with long-term mineral fertilization (HU-min, CONMIN) (Figure 2). In addition, in HUB-LTE soils a significant upregulation of the genes *LOX1*, *WRKY25*, and *MYB15* in shoots of lettuce grown in organically fertilized soil (HU-org) compared to mineral fertilization (HU-min) was observed.

### Chemical Composition of the Rhizosphere Soil Solution

**FIGURE 3:**
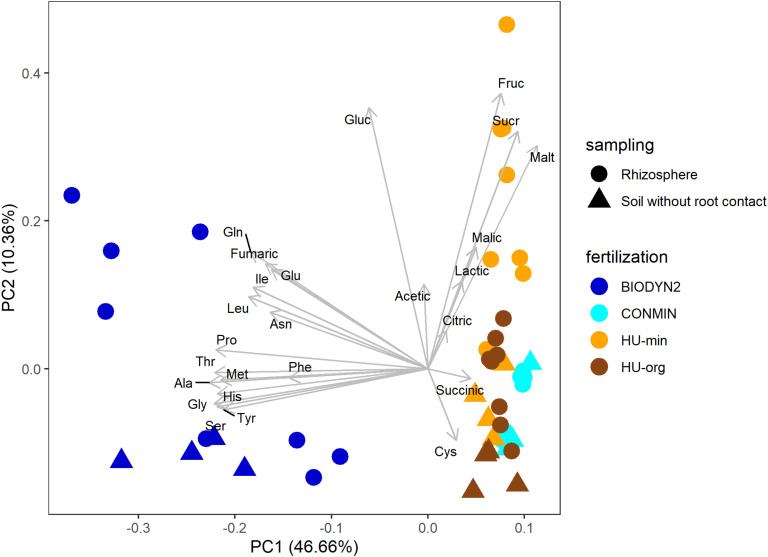
Biplot of PCA comparing patterns of organic compounds in the rhizosphere soil solution of lettuce (cv. Tizian) and in soil without root contact under long-term organic (HU-org, BIODYN2) or mineral (HU-min, CONMIN) fertilization. Ala, alanine; Asn, asparagine; Cys, cysteine; Gln, glutamine; Gly, glycine; Glu, glutamic acid; His, histidine; Ile, iso-leucine; Leu, leucine; Phe, phenylalanine; Pro, proline; Met, methionine; Ser, serine; Thr, threonine; Tyr, tyrosine; Fruc, fructose; Gluc, glucose; Malt, maltose, Sucr, sucrose; Acetic, acetate; Citric, citrate; Fumaric, fumarate; Malic, malate; Succinic, succinate; Lactic, lactate.

Low molecular weight organic compounds in the rhizosphere soil solutions of lettuce grown in the various soil treatments revealed clearly different patterns depending on the fertilization history (Figure 3). The separation of organic compound patterns of organically and minerally fertilized soils were more distinct in DOK-LTE soils compared to HUB-LTE soils. In the different treatments, largely the same organic compounds were detectable with quantitative differences. In the rhizosphere of lettuce grown in BIODYN2 soil but also in BIODYN2 soil without root contact, exceptionally high levels of amino acids were detected (Table 3C and Supplementary Table 3C), leading to separate clustering compared to CONMIN and HUB-LTE soils as shown by PCA analysis (Figure 3). Moreover, higher levels particularly of low molecular weight sugars and malate in the rhizosphere soil solutions collected from lettuce plants grown in HU-min soil explained the separation from HU-org soil (Tables 3A,B and Figure 3).

**TABLE 3:** Sugars **(A)**, carboxylates **(B)**, and amino acids **(C)** in the rhizosphere soil solution of lettuce (cv. Tizian), grown in soils with long-term organic (HU-org, BIODYN2) or mineral (HU-min, CONMIN) fertilization history.

	**HUB-LTE**				**DOK-LTE**			
	**HU-org**	**HU-min**	**HU-org**	**HU-min**	**BIODYN2**	**CONMIN**	**BIODYN2**	**CONMIN**
				
	**Basal (mature)**		**Subapical (young)**		**Basal (mature)**		**Subapical (young)**	
**A. Sugars in the rhizosphere soil solution (nmol cm^–1^ root length)**								
Fructose	2.21 ± 1.04 a	2.02 ± 0.48 a	1.70 ± 0.51 a	4.34 ± 1.31 a	n.d. b	0.93 ± 0.09 a	1.47 ± 0.03 b	2.85 ± 0.03 a
Glucose	n.d. b	1.80 ± 0.49 a	1.62 ± 0.14 a	3.41 ± 0.58 a	n.d. b	1.23 ± 0.18 a	5.95 ± 1.75 a	1.22 ± 0.30 a
Sucrose	0.57 ± 0.06 a	1.53 ± 0.53 a	0.90 ± 0.20 a	1.19 ± 0.31 a	n.d.	n.d.	n.d. b	0.45 ± 0.03 a
Maltose	n.d. b	0.61 ± 0.03 a	0.47 ± 0.01 b	1.00 ± 0.10 a	n.d. b	0.36 ± 0.01 a	n.d. b	0.46 ± 0.02 a

**Sum**	2.79 a	5.97 a	4.70 a	9.95 a	n.d. b	2.54 a	7.43 a	4.97 a

**B. Carboxylates in the rhizosphere soil solution (nmol cm^–1^ root length)**								
Malate	n.d. b	2.91 ± 0.53 a	n.d. b	14.87 ± 3.11 a	n.d.	n.d.	0.72 ± 0.35 a	n.d. b
Citrate	5.42 ± 1.83 a	7.75 ± 3.10 a	2.23 ± 0.57 b	5.73 ± 1.23 a	8.83 ± 3.87 a	3.91 ± 1.46 a	3.37 ± 0.62 a	5.97 ± 1.21 a
Succinate	n.d.	n.d.	n.d.	n.d.	n.d.	n.d.	n.d. b	7.78 ± 1.77 a
Fumarate	n.d. b	0.29 ± 0.05 a	0.30 ± 0.05 a	0.34 ± 0.09 a	0.39 ± 0.07 a	n.d. b	0.56 ± 0.04 a	n.d. b
Benzoate	0.15 ± 0.05 a	0.06 ± 0.01 a	0.10 ± 0.01 a	0.08 ± 0.01 a	0.02 ± 0.005 b	0.11 ± 0.004 a	0.05 ± 0.01 a	0.09 ± 0.01 a

**Sum**	5.57 a	11.02 a	2.63 b	21.03 a	9.25 a	4.02 a	4.71 b	13.85 a

Lactate	59.47 ± 12.41 a	61.83 ± 20.71 a	20.95 ± 4.56 a	61.15 ± 17.22 a	35.83 ± 2.87 a	12.17 ± 3.28 b	35.20 ± 7.68 a	30.96 ± 5.07 a
Acetate	66.65 ± 0.64 a	25.61 ± 11.91 b	11.27 ± 4.99 b	34.20 ± 2.70 a	30.11 ± 21.88 a	11.33 ± 3.93 a	24.52 ± 2.23 a	n.d. b

**Sum**	126.12 a	87.45 a	32.22 b	95.35 a	65.95 a	23.51 a	59.73 a	30.96 a

**C. Amino acids in the rhizosphere soil solution (pmol cm^–1^ root length)**								
Glutamic acid	8 ± 2 a	19 ± 9 a	21 ± 8 a	19 ± 5 a	90 ± 21 a	8 ± 3 b	294 ± 82 a	8 ± 2 b
Asparagine	12 ± 3 a	36 ± 21 a	15 ± 3 a	19 ± 6 a	612 ± 234 a	5 ± 1 b	1868 ± 867 a	6 ± 1 b
Serine	51 ± 6 a	40 ± 11 a	57 ± 5 a	55 ± 2 a	282 ± 18 a	42 ± 4 b	307 ± 14 a	50 ± 6 b
Glutamine	19 ± 10 a	40 ± 14 a	16 ± 6 a	77 ± 35 a	97 ± 16 a	10 ± 3 b	131 ± 9 a	12 ± 2 b
Glycine	56 ± 4 a	36 ± 10 a	48 ± 5 a	49 ± 2 a	249 ± 22 a	41 ± 1 b	290 ± 5 a	42 ± 1 b
Threonine	6 ± 1 a	6 ± 1 a	9 ± 2 a	7 ± 2 a	93 ± 11 a	11 ± 6 b	144 ± 27 a	7 ± 3 b
Histidine	n.d.	n.d.	n.d.	n.d.	15 ± 6 a	n.d. a	26 ± 3 a	n.d. b
Alanine	16 ± 4 a	9 ± 3 a	15 ± 2 a	13 ± 1 a	152 ± 17 a	6 ± 1 b	224 ± 20 a	9 ± 1 b
Proline	10 ± 2 a	6 ± 3 a	17 ± 4 a	18 ± 6 a	51 ± 5 a	6 ± 1 b	70 ± 9 a	7 ± 1 b
Cystine	2 ± 2 a	n.d. a	n.d.	n.d.	n.d.	n.d.	n.d.	n.d.
Thyrosine	n.d.	n.d.	n.d.	n.d.	108 ± 19 a	4 ± 1 b	94 ± 9 a	3 ± 2 b
Methionine	n.d. a	7 ± 1 a	n.d.	n.d.	119 ± 37 a	n.d. b	166 ± 23 a	n.d. b
Isoleucine	n.d. a	7 ± 2 a	n.d. b	8 ± 1 a	n.d.	n.d.	51 ± 15 a	n.d. b
Leucine	n.d. a	0.3 ± 0.3 a	0.4 ± 0.4 b	6 ± 0.4 a	16 ± 0.7 a	n.d. b	88 ± 32 a	n.d. b
Phenylalanine	19 ± 4 a	6 ± 1 b	14 ± 3 a	10 ± 0.6 a	18 ± 2 a	11 ± 2 a	54 ± 13 a	12 ± 3 b

**Sum**	200 a	212 a	212 a	284 a	1903 a	144 b	3808 a	156 b

*The plants were cultivated in minirhizotron culture for nine weeks. Micro-sampling of soil solutions was conducted with sorption filters in 1–2 cm regions of subapical (young) roots and from basal (mature) root zones (8–9 cm). Means ± standard errors. Different lowercase letters indicate significant differences between organic vs. mineral fertilization tested separately for the sites DOK-LTE and HUB-LTE by *t*-test (*p* ≤ 0.05). n.d. = not detectable.*

The rhizosphere soil solution of lettuce was dominated by sugars, carboxylates, and amino acids (Table 3). Among the various mono- and di-saccharides, particularly hexoses, such as glucose and maltose, were present in significantly lower amounts in samples collected from basal (mature) root zones of lettuce plants grown in soils with long-term organic fertilization history (HU-org, BIODYN2) as compared to soils with mineral fertilization (HU-min, CONMIN). In BIODYN2 soil, all sugars ranged below the detection limit (Table 3A).

Lactate and acetate were the dominant monocarboxylates in the rhizosphere soil solutions (Table 3B) but detectable at higher levels also in samples collected from soil without root contact (Supplementary Table 3B). The concentrations of acetate were increased in the rhizosphere of lettuce grown in soils with organic fertilization history (HU-org, BIODYN2) (Table 3B). Among the di- and tri-carboxylates described also in lettuce tissues (Misaghi and Grogan, 1978), citrate was dominant in the rhizosphere soil solution of all treatments. Dicarboxylates such as malate and succinate dominated in soils with long-term mineral fertilization, with particularly high levels of succinate only in subapical (young) root zones in CONMIN soil (Table 3B). The antifungal compound benzoate was found in lower concentrations in the rhizosphere soil solution of older root zones of lettuce plants grown in the BIODYN2 soil compared with all other soils (Table 3B).

### Microbial Community Analyses

#### Site, Habitat, and Fertilization Effects on Microbial Diversity

Alpha-diversity of bacterial and archaeal communities was lower in the rhizosphere than in root-associated soil, when assessed by Shannon, Pielou and richness indices based on 16S rRNA gene sequencing. This effect was more pronounced in HUB-LTE than in DOK-LTE soils. The long-term organic fertilization in DOK-LTE resulted in a significantly higher bacterial and archaeal diversity (Shannon, richness) in both, root-associated soil and the rhizosphere, as compared to mineral fertilization. Evenness, however, was not affected by fertilization in DOK-LTE. Organic fertilization in HUB-LTE also tended to increase the diversity (Shannon, richness) of bacterial and archaeal communities in the rhizosphere and root-associated soil but the effect was not significant (Table 4). Regarding fungal diversity, significantly lower alpha-diversity indices (Shannon, richness, Pielou) in the rhizosphere than in root-associated soil were observed in DOK-LTE based on ITS2 sequencing in both fertilization regimes. In HUB-LTE, this effect was detected only for fungal richness. HU-org significantly increased the fungal species richness compared to HU-min in the rhizosphere and root-associated soil (Shannon, Pielou). A similar trend was observed in the rhizosphere of DOK-LTE soils. No fertilization-dependent effect was observed on fungal evenness.

**TABLE 4:** Microbial alpha-diversity (Shannon diversity, species richness and Pielou’s evenness) in root-associated soil (RA) and rhizosphere (RH) of lettuce (cv. Tizian).

**Diversity Index**	**Habitat**	**Organism**	**HUB-LTE**		**DOK-LTE**	
			**HU-org**	**HU-min**	**BIODYN2**	**CONMIN**
**Microbial alpha-diversity (Shannon diversity, species richness and Pielou’s evenness)**						
Shannon	RA	Bacteria/Archaea	6.68 ± 0.01 Aa	6.73 ± 0.03 Aa	6.42 ± 0.03 Aa	6.33 ± 0.01 Ab
		Fungi	3.33 ± 0.04 Aa	3.39 ± 0.14 Aa	3.39 ± 0.04 Aa	3.37 ± 0.04 Aa
	RH	Bacteria/Archaea	5.29 ± 0.08 Ba	4.49 ± 0.48 Ba	6.29 ± 0.1 Aa	4.67 ± 0.65 Bb
		Fungi	3.36 ± 0.02 Aa	3.07 ± 0.06 Ab	0.68 ± 0.16 Ba	1.12 ± 0.32 Ba

Richness	RA	Bacteria/Archaea	1735.59 ± 12.45 Aa	1723.31 ± 19.86 Aa	1664.18 ± 18.29 Aa	1567.54 ± 8.59 Ab
		Fungi	376.25 ± 2.32 Aa	334.25 ± 7.41 Ab	333.50 ± 9.84 Aa	349.75 ± 5.82 Aa
	RH	Bacteria/Archaea	1007.77 ± 32.82 Ba	829.10 ± 85.15 Ba	1495.93 ± 46.57 Ba	1016.08 ± 140.83 Bb
		Fungi	285.00 ± 8.57 Ba	201.75 ± 8.31 Bb	229.33 ± 20.00 Ba	193.00 ± 14.53 Ba

Pielou	RA	Bacteria/Archaea	0.90 ± 0 Aa	0.90 ± 0 Aa	0.87 ± 0 Aa	0.86 ± 0 Aa
		Fungi	0.56 ± 0.01 Ba	0.58 ± 0.02 Aa	0.58 ± 0 Aa	0.58 ± 0.01 Aa
	RH	Bacteria/Archaea	0.77 ± 0.01 Ba	0.67 ± 0.06 Ba	0.86 ± 0.01 Aa	0.67 ± 0.08 Aa
		Fungi	0.59 ± 0 Aa	0.58 ± 0.01 Aa	0.12 ± 0.03 Ba	0.21 ± 0.06 Ba

*The plants were grown in minirhizotrons for nine weeks in soils with long-term organic (HU-org, BIODYN2) or mineral (HU-min, CONMIN) fertilization history. Data represent means ± standard errors of four independent replicates. Different lowercase letters indicate significant differences between organic vs. mineral fertilization tested separately per site, habitat and organism by *t*-test (*p* ≤ 0.05). Different capital letters indicate significant differences between root-associated soil vs. rhizosphere tested separately per site, fertilization and organism by *t*-test (*p* ≤ 0.05).*

#### Microbial Community Composition Affected by Site, Habitat, and Fertilization History

**FIGURE 4:**
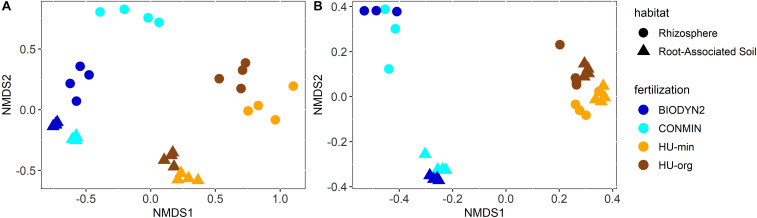
Two-dimensional diagram of non-metric multi-dimensional scaling (NMDS) analysis of calculated Bray-Curtis distances between **(A)** bacterial and archaeal community compositions or **(B)** fungal community compositions in root-associated soil and the rhizosphere of lettuce (cv. Tizian) grown in soils from HUB-LTE (HU-org, HU-min) or DOK-LTE (BIODYN2, CONMIN). NMDS analyses are based on relative abundances. Stress = 0.11 (bacterial and archaeal communities) and 0.07 (fungal communities).

Bacterial and archaeal community composition clearly differed depending on the habitat (*R*^2^ = 30%, *p* < 0.001, PERMANOVA; Figure 4A) and the site (*R*^2^ = 22%, *p* < 0.001). Separate clustering of rhizosphere and root-associated soils was observed for HUB-LTE and DOK-LTE depending on mineral and organic fertilization practice. This finding was confirmed by PERMANOVA analysis which revealed an interaction effect between habitat and site (*R*^2^ = 7%, *p* < 0.001) as well as between habitat and fertilization (*R*^2^ = 3%, *p* ≤ 0.05). A significant influence of the fertilization on the bacterial and archaeal communities was observed (*R*^2^ = 6%, *p* < 0.001). Fertilization-dependent clustering of bacterial and archaeal communities was more distinct in DOK-LTE, especially in the rhizosphere, compared to HUB-LTE (Figure 4A).

The fungal community compositions were clearly separated by site (*R*^2^ = 42%, *p* < 0.001, Figure 4B). Unlike the bacterial and archaeal communities, distinct clustering of rhizosphere and root-associated soils was only observed for DOK-LTE independent of fertilization regimes due to the combined effect of site and habitat (*R*^2^ = 19%, *p* < 0.001). In both LTEs, the impact of fertilization was significant but marginal (*R*^2^ = 5%, *p* < 0.001).

**FIGURE 5:**
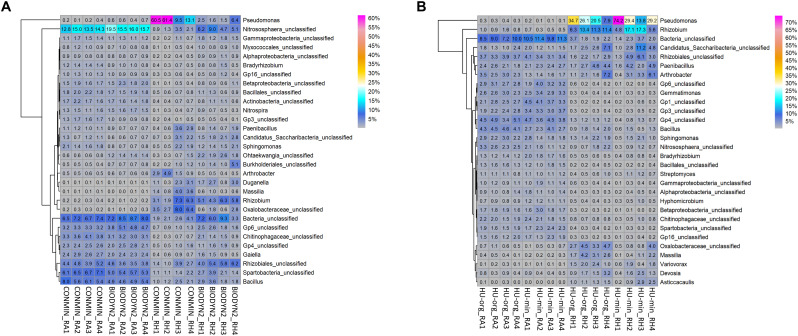
Heatmaps displaying relative abundance distribution of top 30 most abundant bacterial and archaeal genera in root-associated soils (RA) and in the rhizosphere (RH) of lettuce (cv. Tizian) grown in **(A)** DOK-LTE soils (BIODYN2, CONMIN) and **(B)** HUB-LTE soils (HU-org, HU-min). The numbers in cells represent relative abundances (%).

Site, habitat and fertilization effects were also detected in the bacterial and archaeal taxonomic community composition. Thaumarchaeota and Verrucomicrobia had higher relative abundances in DOK-LTE compared to HUB-LTE soils (Supplementary Table 4A). The rhizosphere effect was illustrated by an enrichment in Gamma-, Alpha-, and Betaproteobacteria and by a decrease in relative abundances of Acidobacteria and Firmicutes (Supplementary Table 4A). Heatmap analysis of the most abundant genera in root-associated soils and rhizospheres from HUB-LTE and DOK-LTE showed many taxa occurring at both sites, however most of them exhibited differential relative abundances in rhizosphere and root-associated soils (Figure 5). At both sites, *Bacillus* and *Nitrososphaera* were typical genera found in root-associated soils as well as sequences belonging to the family *Chitinophagaceae* or classified as acidobacterial subdivisions Gp4 and Gp6. Typical rhizosphere responders to lettuce at both sites were affiliated to e.g., *Pseudomonas*, *Rhizobium*, and *Massilia*. When lettuce was grown in HUB-LTE soils, *Variovorax*, *Devosia*, and *Asticcacaulis* were highly abundant in the rhizosphere. In contrast, *Duganella* and sequences classified as *Oxalobacteraceae* were present in the rhizosphere of lettuce grown in DOK-LTE soils. Fertilization affected the relative abundance of major rhizosphere genera. For instance, *Pseudomonas* was highly abundant in the lettuce rhizosphere of CONMIN (up to 61%) and HU-min (up to 74%) and also HU-org (up to 35%). However, a high variability among replicates was observed. In HUB-LTE soils the relative abundance of *Rhizobium* reached up to 13–17% in the rhizosphere, while the remaining major abundant taxa ranged below 10% (Figure 5B).

The root-associated soils of lettuce grown in HUB-LTE were enriched in the fungal phylum Ascomycota, while Mortierellomycota dominated the soils from DOK-LTE independent of fertilization regimes (Supplementary Table 4B). Mineral fertilization increased Basidiomycota at each site. A strong enrichment in Olpidiomycota characterized the rhizosphere in both treatments of DOK-LTE. The rhizosphere in organic fertilization treatments of HUB-LTE also exhibited an increase of this phylum compared to mineral fertilization. Furthermore, Glomeromycota (arbuscular mycorrhizal fungi, AMF) were enriched in the rhizosphere of organic fertilization treatments of both LTEs.

**FIGURE 6:**
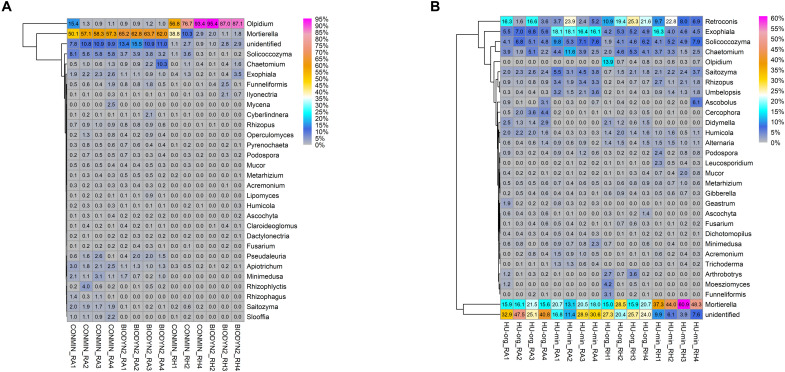
Heatmaps displaying relative abundance distribution of top 30 most abundant fungal genera in root-associated soils (RA) and in the rhizosphere (RH) of lettuce (cv. Tizian) grown in **(A)** DOK-LTE soils (BIODYN2, CONMIN) and **(B)** HUB-LTE soils (HU-org, HU-min). The numbers in cells represent relative abundances (%).

Heatmap analyses of the most abundant fungal genera in root-associated soil and rhizosphere showed that almost half of the genera could be detected in both LTEs but differing in relative abundances (Figure 6). In general, in DOK-LTE more differences were observed between habitats than between fertilization regimes (Figure 6A). The root-associated soils, especially of BIODYN2, were dominated by *Mortierella* and unclassified fungi (at genus level) with a strongly reduced relative abundance in the rhizosphere. Higher relative abundances of different yeasts (*Solicoccozyma, Exophiala, Apiotrichum, Saitozyma*, and *Sloofia*) as well as AMF *Rhizophagus* were observed in minerally fertilized soil (CONMIN). *Olpidium* was the dominant rhizosphere genus in both treatments of DOK-LTE. The differences between the fertilization regimes (organic vs. mineral) were distinct in HUB-LTE (Figure 6B). Typical genera positively affected by organic fertilization independent of habitat were *Cercophora, Didymella*, and *Humicola*. Mineral fertilization (HU-min) enriched not only *Rhizopus* and *Umbelopsis* but also different yeasts (*Exophiala, Solicoccozyma*, and *Saitozyma*), which was in accordance with the DOK-LTE. The rhizosphere of mineral fertilization (HU-min) showed high relative abundances of the genus *Mortierella*, while the rhizosphere of HU-org exhibited a high relative abundance of *Retroconis* and of sequences that could not be reliably classified at genus level.

#### Fertilization Effects on Microbial Communities

As we were interested in the effect of fertilization on plant-microbe interactions, we analyzed fertilization-dependent changes in the relative abundance of microbes in the rhizosphere of lettuce. Taxa differing significantly in relative abundance between organic vs. mineral fertilization were determined separately for each site and at several taxonomic levels [OTU (only for fungi), genus, family, order, class, phylum]. More differentially abundant bacterial and archaeal taxa were found in the rhizosphere of lettuce grown in DOK-LTE soils (>1.0% relative abundance; Table 5). *Pseudomonadaceae* (Gammaproteobacteria) had a significantly higher relative abundance in CONMIN rhizosphere samples compared to BIODYN2 (Table 5B). A similar trend was observed in the rhizosphere of HU-min vs. HU-org, however, differences were not significant. When lettuce was grown in BIODYN2 soil, a significant enrichment in taxa belonging to the Firmicutes phylum (e.g., Clostridiales) was found compared to CONMIN (Table 5B).

**TABLE 5:** Bacterial and archaeal taxa in the rhizosphere of lettuce (cv. Tizian) differing significantly (FDR < 0.05) in relative abundance depending on long-term organic vs. mineral fertilization practice at HUB-LTE (HU-org vs. HU-min) **(A)** and DOK-LTE (BIODYN2 vs. CONMIN) **(B)**.

**Kingdom**	**Phylum**	**Class**	**Order**	**Family**	**Genus**	**HU-org (%)**	**HU-min (%)**
**A. Bacterial rhizosphere taxa differing significantly (FDR < 0.05) in relative abundance in lettuce grown in long-term organically vs. minerally fertilized soils from HUB-LTE**							
Bacteria	Proteobacteria	Gammaproteobacteria	Pseudomonadales	*Pseudomonadaceae*	*Cellvibrio*	**1.1 ± 0.4**	0.0 ± 0.0
Bacteria	Proteobacteria	Betaproteobacteria	Methylophilales	*Methylophilaceae*	*Methylophilus*	**1.2 ± 0.7**	0.0 ± 0.0

**Kingdom**	**Phylum**	**Class**	**Order**	**Family**	**Genus**	**BIODYN2 (%)**	**CONMIN (%)**

**B. Bacterial and archaeal rhizosphere taxa differing significantly (FDR < 0.05) in relative abundance in lettuce grown in long-term organically vs. minerally fertilized soils from DOK-LTE**							
Archaea	Thaumarchaeota					**6.3 ± 1.0**	1.9 ± 0.6
Bacteria	Proteobacteria	Alphaproteobacteria	Sphingomonadales	*Sphingomonadaceae*	*Sphingomonadaceae*_unclassified	**1.1 ± 0.1**	0.2 ± 0.1
Bacteria	Proteobacteria	Betaproteobacteria	Burkholderiales	Burkholderiales_incertae_sedis		**1.1 ± 0.3**	0.1 ± 0.0
Bacteria	Firmicutes	Clostridia				**3.5 ± 0.7**	0.8 ± 0.2
Bacteria	Firmicutes	Clostridia	Clostridiales			**3.3 ± 0.7**	0.8 ± 0.2
Bacteria	Firmicutes	Firmicutes_unclassified				**1.0 ± 0.2**	0.0 ± 0.0
Bacteria	Firmicutes	Firmicutes_unclassified	Firmicutes_unclassified			**1.0 ± 0.2**	0.0 ± 0.0
Bacteria	Firmicutes	Firmicutes_unclassified	Firmicutes_unclassified	Firmicutes_unclassified		**1.0 ± 0.2**	0.0 ± 0.0
Bacteria	Firmicutes	Firmicutes_unclassified	Firmicutes_unclassified	Firmicutes_unclassified	*Firmicutes*_unclassified	**1.0 ± 0.2**	0.0 ± 0.0
Bacteria	Cyanobacteria/Chloroplast					**1.2 ± 0.7**	0.0 ± 0.0
Bacteria	Cyanobacteria/Chloroplast	Cyanobacteria				**1.2 ± 0.7**	0.0 ± 0.0
Bacteria	Actinobacteria					7.3 ± 0.8	**8.2 ± 0.8**
Bacteria	Actinobacteria	Actinobacteria				6.9 ± 0.8	**7.9 ± 0.7**
Bacteria	Actinobacteria	Actinobacteria	Actinomycetales			4.3 ± 0.4	**6.5 ± 0.7**
Bacteria	Actinobacteria	Actinobacteria	Actinomycetales	*Micrococcaceae*		0.5 ± 0.0	**3.2 ± 0.9**
Bacteria	Actinobacteria	Actinobacteria	Actinomycetales	*Micrococcaceae*	*Arthrobacter*	0.5 ± 0.0	**2.6 ± 0.9**
Bacteria	Proteobacteria					46.6 ± 4.7	**69.3 ± 5.3**
Bacteria	Proteobacteria	Betaproteobacteria	Burkholderiales	*Oxalobacteraceae*		4.2 ± 1.1	**9.7 ± 2.5**
Bacteria	Proteobacteria	Betaproteobacteria	Burkholderiales	*Oxalobacteraceae*	*Massilia*	0.6 ± 0.1	**2.5 ± 0.8**
Bacteria	Proteobacteria	Gammaproteobacteria				8.6 ± 1.4	**39.8 ± 13.4**
Bacteria	Proteobacteria	Gammaproteobacteria	Pseudomonadales			5 ± 1.2	**37.3 ± 14.1**
Bacteria	Proteobacteria	Gammaproteobacteria	Pseudomonadales	*Pseudomonadaceae*		4.9 ± 1.2	**36.9 ± 14.1**

*Only taxa with >1.0% relative abundance are displayed. Data represent means of relative abundance ± standard errors. Bold numbers indicate significant enrichment.*

In contrast to bacteria and archaea, more differentially abundant fungal taxa were found in the rhizosphere of lettuce grown in HUB-LTE soils compared to DOK-LTE (Table 6). To obtain further insights into the ecological assignment of detected fungal genera, they were assessed against the FUNGuild database for classification into potential pathotrophic, saprotrophic or symbiotrophic fungi (Supplementary Table 5). In concordance with heatmaps (Figure 6), the potential pathotrophic genus *Olpidium* showed the highest relative abundance in the rhizosphere of lettuce when grown in both DOK-LTE soils and was enriched in the root-associated soil of CONMIN (Supplementary Tables 5, 6B). The rhizosphere of HU-org showed the highest number of significantly enriched pathotrophs including the genera *Olpidium, Moesziomyces*, and *Ascochyta* whereas the root-associated soil of HU-min was enriched with pathotrophic-saprotrophic fungi, especially *Exophiala*. The plant pathogen *Rhizopus* was increased in the root-associated soil and in the rhizosphere of HU-min, whereas *Didymella* was increased in the root-associated soil and in the rhizosphere of HU-org. The saprotrophic genera *Cercophora* and *Humicola* as well as *Arthrobotrys* and *Plenodomus* were highly abundant in the root-associated soil and in the rhizosphere of HU-org, respectively. *Umbelopsis* was enriched in both habitats of HU-min (Table 6A and Supplementary Table 6A). Significantly more sequences classified as *Mortierella*, a saprotrophic-symbiotrophic genus, were found in the rhizosphere of lettuce grown in minerally fertilized soils (HUB-LTE and DOK-LTE) in comparison with organic fertilization. Mycorrhizal symbiotrophs (*Clariodeoglomus, Funneliformis*) were enriched in both habitats of lettuce grown in HU-org whereas the relative abundance of *Trichoderma* increased in root-associated soil of HU-min (Supplementary Table 5). Further results for fungal taxa in the root-associated soils are shown in Supplementary Table 6.

**TABLE 6:** Relative abundance of fungal taxa in the rhizosphere of lettuce (cv. Tizian) differing significantly (FDR < 0.05) in relative abundance depending on long-term organic vs. mineral fertilization practice at HUB-LTE (HU-org vs. HU-min) **(A)** and DOK-LTE (BIODYN2 vs. CONMIN) **(B)**.

**Phylum**	**Class**	**Order**	**Family**	**Genus**	**OTU**	**HU-org (%)**	**HU-min (%)**
**A. Fungal rhizosphere taxa differing significantly (FDR < 0.05) in relative abundance of lettuce grown in long-term organically vs. minerally fertilized soils from HUB-LTE**							
Ascomycota	Dothideomycetes					**16.2 ± 1.6**	2.7 ± 0.4
Ascomycota	Dothideomycetes	Pleosporales				**16.1 ± 1.6**	2.7 ± 0.4
Ascomycota	Dothideomycetes	Pleosporales	*Didymellaceae*			**13.2 ± 1.5**	0.8 ± 0.3
Ascomycota	Dothideomycetes	Pleosporales	*Didymellaceae*	*Didymella*		**1.4 ± 0.3**	0 ± 0
Ascomycota	Dothideomycetes	Pleosporales	*Didymellaceae*	*Didymella*	*Didymella protuberans* (100%)	**1.3 ± 0.3**	0 ± 0
Ascomycota	Dothideomycetes	Pleosporales	*Didymellaceae*	unidentified	*Didymellaceae* sp.	**11.3 ± 1.1**	0.8 ± 0.3
Ascomycota	Orbiliomycetes					**1.8 ± 0.8**	0.1 ± 0
Ascomycota	Orbiliomycetes	Orbiliales				**1.8 ± 0.8**	0.1 ± 0
Ascomycota	Orbiliomycetes	Orbiliales	*Orbiliaceae*			**1.8 ± 0.8**	0.1 ± 0.1
Ascomycota	Orbiliomycetes	Orbiliales	*Orbiliaceae*	*Arthrobotrys*		**1.8 ± 0.8**	0.1 ± 0.1
Ascomycota	Orbiliomycetes	Orbiliales	*Orbiliaceae*	*Arthrobotrys*	*Arthrobotrys oligospora* (100%)	**1.6 ± 0.8**	0.1 ± 0.1
Ascomycota	Pezizomycetes	Pezizales	*Pezizaceae*			**2.2 ± 2.1**	0 ± 0
Ascomycota	Pezizomycetes	Pezizales	*Pezizaceae*	unidentified	*Pezizaceae* sp.	**2.2 ± 2.1**	0 ± 0
Basidiomycota	Agaricomycetes					**1.5 ± 0.4**	0.3 ± 0.1
Basidiomycota	Ustilaginomycetes					**1.2 ± 1.0**	0 ± 0
Basidiomycota	Ustilaginomycetes	Ustilaginales				**1.2 ± 1.0**	0 ± 0
Basidiomycota	Ustilaginomycetes	Ustilaginales	*Ustilaginaceae*			**1.2 ± 1.0**	0 ± 0
Basidiomycota	Ustilaginomycetes	Ustilaginales	*Ustilaginaceae*	*Moesziomyces*		**1.2 ± 1.0**	0 ± 0
Basidiomycota	Ustilaginomycetes	Ustilaginales	*Ustilaginaceae*	*Moesziomyces*	*Moesziomyces aphidis* (100%)	**1.2 ± 1.0**	0 ± 0
Glomeromycota						**1.8 ± 1.1**	0 ± 0
Glomeromycota	Glomeromycetes					**1.8 ± 1.1**	0 ± 0
Glomeromycota	Glomeromycetes	Glomerales				**1.8 ± 1.1**	0 ± 0
Glomeromycota	Glomeromycetes	Glomerales	*Glomeraceae*			**1.0 ± 0.8**	0 ± 0
Olpidiomycota						**4.0 ± 3.3**	0.5 ± 0.1
Olpidiomycota	Olpidiomycetes					**4.0 ± 3.3**	0.5 ± 0.1
Olpidiomycota	Olpidiomycetes	Olpidiales				**4.0 ± 3.3**	0.5 ± 0.1
Olpidiomycota	Olpidiomycetes	Olpidiales	*Olpidiaceae*			**4.0 ± 3.3**	0.5 ± 0.1
Olpidiomycota	Olpidiomycetes	Olpidiales	*Olpidiaceae*	*Olpidium*		**4.0 ± 3.3**	0.5 ± 0.1
Olpidiomycota	Olpidiomycetes	Olpidiales	*Olpidiaceae*	*Olpidium*	*Olpidium brassicae* (99.7%)	**4.0 ± 3.3**	0.5 ± 0.1
–	−	−	−	unidentified		**24.3 ± 1.5**	6.9 ± 1.3
Ascomycota	Pezizomycetes	Pezizales	*Ascobolaceae*			0.2 ± 0.1	**1.6 ± 1.5**
Ascomycota	Sordariomycetes	Sordariales	*Lasiosphaeriaceae*	*Podospora*		0.2 ± 0	**1.1 ± 0.5**
Ascomycota	Sordariomycetes	Sordariales	*Lasiosphaeriaceae*	*Podospora*	*Podospora* sp.	0.2 ± 0	**1.0 ± 0.5**
Basidiomycota	Tremellomycetes	Tremellales				1.5 ± 0.3	**2.6 ± 0.4**
Mortierellomycota						20.0 ± 3.1	**47.8 ± 5.0**
Mortierellomycota	Mortierellomycetes					20.0 ± 3.1	**47.8 ± 5.0**
Mortierellomycota	Mortierellomycetes	Mortierellales				20.0 ± 3.1	**47.8 ± 5.0**
Mortierellomycota	Mortierellomycetes	Mortierellales	Mortierellaceae			20.0 ± 3.1	**47.6 ± 5.0**
Mortierellomycota	Mortierellomycetes	Mortierellales	Mortierellaceae	*Mortierella*		20.0 ± 3.1	**47.6 ± 5.0**
Mortierellomycota	Mortierellomycetes	Mortierellales	Mortierellaceae	*Mortierella*	*Mortierella humilis* (100%)	0 ± 0	**1.7 ± 0.6**
Mortierellomycota	Mortierellomycetes	Mortierellales	Mortierellaceae	*Mortierella*	*Mortierella hyalina* (100%)	1.1 ± 0.3	**8.3 ± 4.0**
Mortierellomycota	Mortierellomycetes	Mortierellales	Mortierellaceae	*Mortierella*	*Mortierella* sp.	1.1 ± 0.2	**4.2 ± 1.1**
Mucoromycota						1.0 ± 0.2	**4.3 ± 0.5**
Mucoromycota	Mucoromycetes					0.8 ± 0.2	**2.9 ± 0.6**
Mucoromycota	Mucoromycetes	Mucorales				0.8 ± 0.2	**2.9 ± 0.6**
Mucoromycota	Mucoromycetes	Mucorales	Rhizopodaceae			0.5 ± 0.1	**1.9 ± 0.3**
Mucoromycota	Mucoromycetes	Mucorales	Rhizopodaceae	*Rhizopus*		0.5 ± 0.1	**1.9 ± 0.3**
Mucoromycota	Mucoromycetes	Mucorales	Rhizopodaceae	*Rhizopus*	*Rhizopus arrhizus* (100%)	0.5 ± 0.1	**1.9 ± 0.3**
Mucoromycota	Umbelopsidomycetes					0.2 ± 0	**1.4 ± 0.2**
Mucoromycota	Umbelopsidomycetes	Umbelopsidales				0.2 ± 0	**1.4 ± 0.2**
Mucoromycota	Umbelopsidomycetes	Umbelopsidales	Umbelopsidaceae			0.2 ± 0	**1.4 ± 0.2**
Mucoromycota	Umbelopsidomycetes	Umbelopsidales	Umbelopsidaceae	*Umbelopsis*		0.2 ± 0	**1.4 ± 0.2**
Mucoromycota	Umbelopsidomycetes	Umbelopsidales	Umbelopsidaceae	*Umbelopsis*	*Umbelopsis* sp.	0.2 ± 0	**1.4 ± 0.2**

**Phylum**	**Class**	**Order**	**Family**	**Genus**	**OTU**	**BIODYN2 (%)**	**CONMIN (%)**

**B. Fungal rhizosphere taxa differing significantly (FDR < 0.05) in relative abundance of lettuce grown in long-term organically vs. minerally fertilized soils from DOK-LTE**							
Glomeromycota						**1.5 ± 0.9**	0.2 ± 0
Glomeromycota	Glomeromycetes	Glomerales				**1.4 ± 0.9**	0.1 ± 0
Basidiomycota	Tremellomycetes	Filobasidiales	*Piskurozymaceae*			0.1 ± 0	**1.7 ± 1.0**
Mortierellomycota						1.6 ± 0.3	**17.3 ± 11.0**
Mortierellomycota	Mortierellomycetes	Mortierellales	*Mortierellaceae*			1.6 ± 0.3	**17.3 ± 11.0**
Mortierellomycota	Mortierellomycetes	Mortierellales	*Mortierellaceae*	*Mortierella*	*Mortierella elongata* (100%)	0.2 ± 0	**7.2 ± 5.7**

*Only taxa with >1.0% relative abundance are displayed. For OTUs at species level, the percentages of similarity compared to the database are specified. Data represent means of relative abundance ± standard errors. Bold numbers indicate significant enrichment.*

**FIGURE 7:**
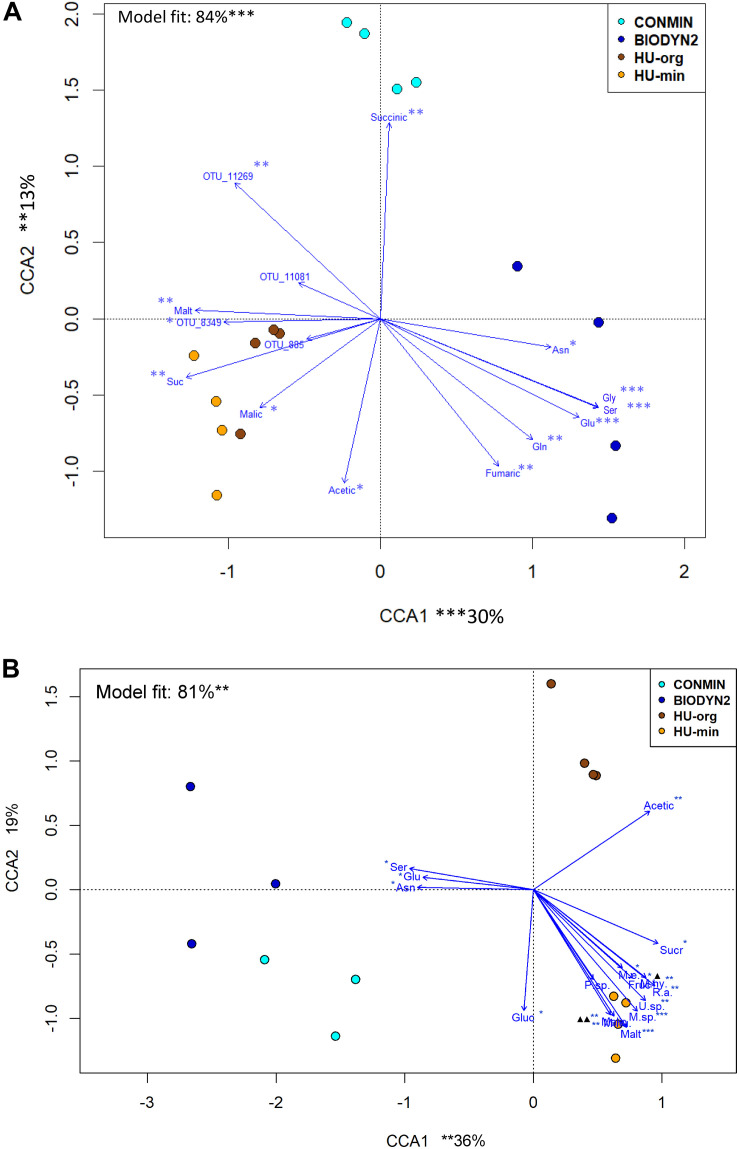
Canonical correspondence analysis (CCA) of bacterial and archaeal **(A)** and fungal **(B)** community composition in the rhizosphere of lettuce grown in soils with long-term organic (HU-org, BIODYN2) or mineral (HU-min, CONMIN) fertilization. CCA is based on log10 transformed relative abundances of bacterial, archaeal and fungal OTUs using organic compounds in rhizosphere soil solution averaged over basal (mature) and subapical root zones. **(A)** Bacterial and archaeal communities: Acetate (Acetic) + malate (Malic) + succinate (Succinic) + fumarate (Fumaric) + maltose (Malt) + sucrose (Suc) + asparagine (Asn) + serine (Ser) + glutamine (Gln) + glutamic acid (Glu) + glycine (Gly) as constraint variables. Significant vectors were fitted onto CCA ordination including log transformed relative abundances of *Pseudomonadaceae* OTUs that were significantly enriched (FDR < 0.05) in minerally fertilized soils and exhibited a mean relative abundance of >0.5%. Tentative taxonomic identification of OTUs based on most similar BLAST hit: OTU_11269 – *Pseudomonas corrugata* (99.77%); OTU_11081 – *P. silesiensis* (96.97%); OTU_8349 – *P. corrugata* (99.3%); OTU_885 – *P. tolaasii* (99.77%). Significant codes: ^∗^*p* < 0.05, ^∗∗^*p* < 0.01, ^∗∗∗^*p* < 0.001. **(B)** Fungal communities: Acetate (Acetic) + malate (Malic) + fructose (Fruc) + glucose (Gluc) + sucrose (Sucr) + maltose (Malt) + glutamic acid (Glu) + asparagine (Asn) + serine (Ser) as constraint variables. Significant vectors were fitted onto CCA ordination including log transformed relative abundances of OTUs (identified at least at genus level) that were significantly enriched (FDR < 0.05) in minerally fertilized soils and exhibited a mean relative abundance of >1.0% (M.e. – *Mortierella elongata*; R.a. – *Rhizopus arrhizus*; M.sp. – *Mortierella* sp.*;* P.sp. − *Podospora* sp.; U.sp. – *Umbelopsis* sp.; M.hy. – *Mortierella hyalina*; M.hu. – *Mortierella humilis*). Overlapping designations: ▲ Fruc^∗^/M.e.^∗^/M.hy.^∗∗^, ▲▲ Malic^∗∗^/M.hu.^∗∗^. Significant codes: ^∗^*p* < 0.05, ^∗∗^*p* < 0.01, ^∗∗∗^*p* < 0.001.

Canonical correspondence analyses revealed relationships in rhizosphere bacterial and archaeal and fungal community composition with organic compounds detected in the rhizosphere soil solution (Figure 7). Ordination showed that the fertilization-dependent differentiation in rhizosphere bacterial and archaeal communities of lettuce grown in DOK-LTE soils was clearly related to the concentration of succinate (CONMIN) or fumarate and amino acids (BIODYN2). HUB soils were positively associated with sugars (sucrose, maltose) which caused the separate clustering of rhizosphere bacterial and archaeal communities apart from DOK-LTE soil samples. Fertilization-dependent community differences in HUB-LTE soils were less clear as compared to DOK-LTE soils but a significant contribution of malate to the differentiation of HU-min was identified. The relative abundances of *Pseudomonadaceae* OTUs responding positively to mineral fertilization were negatively correlated with fumaric and amino acids (Figure 7A).

Fertilization-dependent differences of fungal rhizosphere communities were more pronounced in HUB-LTE compared to DOK-LTE (Figure 7B). The clear separation between organic vs. mineral fertilization (HUB-LTE) was caused by the positive correlation of several compounds in HU-min compared to only one in HU-org (acetate). Fungal communities of lettuce grown in HU-min soils showed a clear association to sugars (sucrose, maltose, and fructose) and malate, which was partly comparable with the bacterial and archaeal communities. The identified responder OTUs (*Rhizopus arrhizus, Mortierella* sp., *Umbelopsis* sp., *Mortierella hyalina*, *Mortierella humilis*, and *Nigrospora oryzae*, Table 6) reacted positively to mineral fertilization. Regarding all four treatments, *M. elongata* responded also positively to mineral fertilization in HUB-LTE, although this OTU was significantly enriched in CONMIN compared to BIODYN2 (Table 6B). Comparable with bacterial and archaeal communities, fungal rhizosphere communities of lettuce grown in DOK soils, especially BIODYN2, were clearly associated with amino acids.

## Discussion

Plant roots are described as powerful drivers of microbiota assemblage (Bais et al., 2006; Bakker et al., 2014). However, to which extent site-specific factors or fertilization management interact with the recruitment of rhizosphere microbiota remains largely unclear. In a holistic approach, we tried to correlate the structure of rhizosphere microbial communities with data on organic composition of the rhizosphere soil solution, root growth characteristics and aboveground plant traits (biomass, nutrient status and expression of stress-related genes). Two strategies of fertilization management were exemplarily compared by growing lettuce plants in minirhizotrons with contrasting soils from two LTEs with organic and mineral fertilization history.

### Site- and Fertilization-Dependent Plant Performance

No consistent effects of long-term fertilization practice on aboveground plant biomass and root characteristics (total and fine root length, average diameter, and length of root hairs) were detected in this study (Figure 1), as similarly reported in an earlier lettuce experiment with the same soils (Chowdhury et al., 2019). The latter authors reported similar growth in DOK-LTE soils and lower plant biomass in organically fertilized soil of the HUB-LTE (Chowdhury et al., 2019). In our experiment, the results were opposite, with similar biomass in soils of the HUB-LTE and shoot and root biomass drastically reduced by 41% and 81%, respectively, in the organically fertilized soil (BIODYN2) of the DOK-LTE. This effect could not be attributed to nutrient limitations. Although at the end of the experiment, concentrations of mineral nutrients (N, P, K) in shoot tissues were below or close to the reported deficiency thresholds (Bergmann, 1988), these effects were observed similarly for all soil treatments and not only for the BIODYN2 soil (Table 2). Similar plant growth reductions were observed in a repeated experiment with the same soils, already detectable during early plant establishment (Supplementary Table 2). This suggests the presence of additional stress factors in BIODYN2 soil independent of nutrient limitations, as a site-specific effect. The discrepancy between our findings and those of Chowdhury et al. (2019) may have resulted from the collection of the soils in different years with different pre-crops. Effects of the pre-crop on soil microbial communities have been reported previously (Sommermann et al., 2018; Babin et al., 2019) and the detritusphere microbiome of pre-crop roots can even overwrite the rhizosphere effect of the current crop (Zhou et al., 2020) with potential consequences for plant performance. Furthermore, in the study of Chowdhury et al. (2019) lettuce plants were cultivated in pots, whereas our study was conducted with minirhizotrons promoting the development of high rooting densities along the root observation windows. This may lead to locally increased exudate concentrations contributing to attraction of beneficial but also of pathogenic microorganisms with potential impact on plant performance.

### Soil Microbial Communities Affected by Fertilization, Site, and Habitat Effects

The LTE site and the habitats, comprising rhizosphere and root-associated soil, distinguished bacterial, archaeal, and fungal community composition as recently reported by Chowdhury et al. (2019). A significantly higher bacterial and archaeal alpha-diversity was mainly found in the rhizosphere of lettuce when grown in organically vs. minerally fertilized soils of DOK-LTE but not of HUB-LTE (Table 4). Conversely, the alpha-diversity of the fungal rhizosphere community was significantly increased in organically fertilized soil of HUB-LTE, but not of DOK-LTE, illustrating the impact of the soil type as a major driver determining not only the soil but also the rhizosphere microbial composition (Schreiter et al., 2014b; Chowdhury et al., 2019). The selective effect of the plant on recruitment of microbial communities was apparent by a lower alpha-diversity in the rhizosphere compared to root-associated soil (Table 4) as reported in various other studies (Mendes et al., 2013; Chowdhury et al., 2019).

The majority of bacterial and archaeal rhizosphere responders were classified as Gammaproteobacteria of the genus *Pseudomonas* with particularly high relative abundance (up to 60–74%) in soils with mineral fertilization history, while only marginal rhizosphere enrichment (rel. abundance 2–6%) was detectable in the BIODYN2 rhizosphere with long-term organic fertilization (Figure 5). Many members of this genus exert beneficial effects on plants (Roquigny et al., 2017), among them *Pseudomonas putida*, *Pseudomonas fluorescens*, and *Pseudomonas brassicacearum*, although pathogens are also reported. Since we observed the lowest biomass production along with impaired fine root development in lettuce plants grown in the BIODYN2 soil (Figure 1B), this may indicate a relationship between the low relative abundance of potentially beneficial *Pseudomonadaceae* and plant growth, because lettuce growth was not impaired in the soils with a particularly high *Pseudomonas* relative abundance (Figures 1, 5). A preliminary identification showed high similarities with members of the *P. fluorescens* complex with plant growth-promoting properties (Garrido-Sanz et al., 2016). However, strain identification based on short-read Illumina 16S rRNA gene sequences is limited. Future studies should therefore consider cultivation-based approaches for comprehensive taxonomic and functional characterization.

With regard to fungal communities, the most remarkable rhizosphere effect was the enrichment of the pathogenic genus *Olpidium* in the rhizosphere of soils with organic fertilization history (HU-org, BIODYN2), which was particularly pronounced for DOK-LTE, where its relative abundance reached 76-90% (Figure 6 and Supplementary Table 4B). Lettuce is a host for *Olpidium* sp. (Olpidiomycota, formerly classified as Chytridiomycota). Zoospores of the fungus with random motility rapidly invade the tips of fine roots (Maccarone, 2013). Since these zoospores are not specifically attracted by root exudates (Westerlund et al., 1978), direct relationships with the observed site- and fertilization-dependent differences in the composition of the rhizosphere soil solution (Figure 3 and Table 3) are unlikely. The pathogen can survive in soils as dormant spores for up to 20 years (Campbell, 1985). Early plant infection can result in severe growth inhibition (Navarro et al., 2004). Accordingly, in our study, growth retardation and inhibition of root hair development in the BIODYN2 soil (Figure 1B and Supplementary Table 2) was associated with typical symptoms of *Olpidium* infection. Root infection with intracellular formation of sporangia and resting spores in fine roots (Supplementary Figure 1) was consistent with the highest relative abundance of *Olpidium* (90%) in the BIODYN2 rhizosphere (Figure 6 and Supplementary Table 5). Interestingly, in the CONMIN soil, symptoms caused by *Olpidium* were less pronounced despite similarly high relative *Olpidium* abundance in the rhizosphere of both treatments CONMIN (76%) and BIODYN2 soil (90%) (Supplementary Table 5). Zoospores exhibit high motility in wet clay soils to initiate infections in nearby plants (Westerlund et al., 1978). This may explain the low relative abundance of *Olpidium* in the sandy HUB-LTE soils (0.5–4%) and increased infection rates in the silty loam DOK-LTE soils with a higher water retention capacity as a site-specific effect. Nevertheless, this cannot explain the high prevalence of disease symptoms in plants from BIODYN2 soil (Supplementary Figure 1) with organic fertilization compared to CONMIN soil supplied with mineral fertilizers despite similar relative *Olpidium* abundance in the rhizosphere (Supplementary Table 5 and Figure 6).

Along with the rhizosphere enrichment in *Olpidium*, a particularly low relative abundance of the fungal genus *Mortierella* (1.6%) was found for the BIODYN2 rhizosphere, reaching 17-48% in the other investigated treatments (Figure 6 and Supplementary Table 5). Fungal endophytes of the genus *Mortierella* are classified as saprotrophic symbiotrophs (Supplementary Table 5). Most recently, plant growth-promoting properties and biocontrol activity against fungal pathogens via jasmonic acid-dependent plant stress signaling were reported for various *Mortierella* species (Kamzolova et al., 2014; Tamayo-Velez and Osorio, 2017; Li et al., 2018) including *Mortierella elongata* identified in our study (Table 6B). Therefore, we suggest that higher sensitivity toward *Olpidium* infection of lettuce plants grown in the BIODYN2 soil may be a consequence of a low relative abundance of bacterial (*Pseudomonas*) and fungal (*Mortierella*) antagonists or plant growth promoters in the rhizosphere. In contrast, a selective pathogen-suppressive effect of long-term pesticide application in the CONMIN soil (Table 1) seems to be unlikely in this case, since the relative *Olpidium* abundance was even higher in the root-associated CONMIN soil compared to BIODYN2 soil (Supplementary Table 5).

Apart from rhizosphere enrichment of potential plant pathotrophs and saprotrophs, AMF (*Funneliformis* and *Claroideoglomus*) as plant beneficial symbiothrophs, increased in organically fertilized soils (BIODYN2 and HU-org). A higher extent of AMF root colonization and increased AMF species diversity in organic farming soils has been frequently reported (Sattelmacher et al., 1991; Douds et al., 1997; Gosling et al., 2006) as a consequence of lower P availability (Nagahashi et al., 1996; Olsson et al., 2003) or reduced fertilizer and pesticide inputs. Accordingly, the lowest P-nutritional status was recorded for lettuce plants grown in BIODYN2 and HU-org soils (Table 2).

### Components of the Rhizosphere Soil Solution Related to Rhizosphere Microbiota

In our study, significantly lower sugar concentrations (particularly glucose) were recorded in the rhizosphere soil solution of mature roots of lettuce plants grown in soils with organic compared to mineral fertilization history (Table 3A). Host plants provide up to 20% of photo-assimilates to mature arbuscular-mycorrhizal root systems, consumed preferentially in form of glucose by AMF. Consequently, this is frequently associated with lower sugar exudation into the rhizosphere (Jones et al., 2004). Accordingly, also in our study, lower glucose concentrations in the lettuce rhizosphere in soils with long-term organic fertilization coincided with a higher rhizosphere relative abundance of AMF (Table 6 and Supplementary Table 4B). Interestingly, in lettuce plants grown in BIODYN2 soil, with the highest root infection rate by the *Olpidium* pathogen (Figure 6 and Supplementary Table 5), sugar concentrations ranged close to or even below the detection limit in the rhizosphere of mature root zones (Table 3A). This is most likely caused by the high carbon demand and sugar consumption of the obligate biotrophic pathogen. On the other hand, some reports show antagonistic effects of AMF colonization against *Olpidium* (Hao et al., 2019) but it remains questionable, whether this applies for competitive root colonization of *Olpidium* and AMF in the establishment phase during early growth of the host plants analyzed in our study. The extremely limited availability of glucose in the rhizosphere of the BIODYN2 plants (Table 3A) may also explain the particularly low relative rhizosphere abundance of *M. elongata* (Table 6B) with potential antagonistic properties (Li et al., 2018), since glucose supply was identified as a major carbon source for stimulation of *Mortierella* mycelium growth (Chesters and Peberdy, 1965). Noteworthy, also antagonistic effects between root colonization with AMF and *Mortierella* sp. were recently reported in avocado nurseries (Tamayo-Velez and Osorio, 2017). This might at least partially explain the higher *Mortierella* rhizosphere relative abundance in the soils with long-term mineral fertilization history (Table 6 and Supplementary Table 4B).

Windisch et al. (2017) reported the ability of bacterial biocontrol inoculants (*Pseudomonas* sp. RU47; *Serratia plymuthica* 3Re-4-18) to induce root exudation of benzoate in lettuce, as a secondary metabolite with antifungal activity (Yoon et al., 2012). This was related to increased tolerance against bottom rot disease caused by *Rhizoctonia solani*. Surprisingly, in our study, the significantly reduced rhizosphere relative abundance of *Pseudomonaceae* in lettuce plants grown in the BIODYN2 soil in comparison with the CONMIN soil (Table 5 and Figure 5) was associated with *Olpidium*-related growth depressions and significantly reduced benzoate concentrations in the rhizosphere soil solution (Table 3B). Therefore, a low rhizosphere presence of bacterial communities with antagonistic potential in the BIODYN2 soil (e.g., *Pseudomonas*) may be responsible for the limited stimulation of benzoate exudation, thereby increasing the plant sensitivity to pathogen attack. Moreover, benzoate also has chemo-attractive properties for various *Pseudomonas* species (reviewed by Sampedro et al., 2015).

In terms of di- and tri-carboxylates, characterized as intracellular compounds in lettuce (Misaghi and Grogan, 1978), high concentrations of succinate were detected exclusively in the rhizosphere soil solution of lettuce grown in the CONMIN soil (Table 3B) and related to a high relative abundance of *Pseudomonadaceae* (Figure 7A). Among other dicarboxylates, such as malate and fumarate, also detected in the rhizosphere soil solutions of the investigated lettuce plants particularly in combination with mineral fertilization (Table 3B), succinate has been characterized as a potent chemoattractant with importance for root colonization by various *Pseudomonas* strains with plant growth-promoting properties (Oku et al., 2014; reviewed by Sampedro et al., 2015). Moreover, succinate is the major carbon source required for production of pyoverdine siderophores by fluorescent pseudomonads (Marek-Kozaczuk and Skorupska, 1997) with biocontrol functions via iron competition with pathogens (Höfte et al., 1991) and induction of systemic plant defense responses (Audenaert et al., 2002; Adesina et al., 2007). Therefore, we speculate that a potential relationship between the enrichment of beneficial *Pseudomonadaceae* and high succinate concentrations in the CONMIN rhizosphere counteracted *Olpidium* pathogenesis.

Additionally, reduced sugar concentrations in connection with extremely high amino acid concentrations resulted in a lower C/N ratio in the rhizosphere soil solution of plants grown in the BIODYN2 soil, as compared with all other soil treatments (Table 3). Gammaproteobacteria are generally characterized as fast-growing, copiotrophic microorganisms, which prefer carbon-rich environments and hence a preference for high C/N substrate ratios has been described for Gammaproteobacteria (Kuramae et al., 2012; Michaud et al., 2014; Shen et al., 2016). Consequently, the relatively high N and low C availability in the rhizosphere of lettuce grown in BIODYN2 soil may have counteracted an enrichment of Gammaproteobacteria (i.e., *Pseudomonadaceae*) with potentially antagonistic properties in the rhizosphere. Accordingly, amino acid accumulation in the rhizosphere was negatively correlated with rhizosphere relative abundance of *Pseudomonadaceae* (Figure 7A). Similarly high amino acid concentrations were recorded also in samples collected from BIODYN2 soil without root contact (Supplementary Table 3C). A possible explanation for this phenomenon is the high organic N content potentially affected by long-term input of composted farmyard manure in BIODYN2 soil with the highest C_*org*_ and total N contents among all other investigated soils (Table 1B). Thus, a high mineralization potential and elevated levels of free amino acids as intermediate products may have led to the observed increase of amino acid concentrations in the soil solution largely overwriting the rhizosphere effect. Nevertheless, a wide range of amino acids has been reported as chemoattractants for *Pseudomonas* strains (reviewed by Sampedro et al., 2015) but due to the absence of a rhizosphere effect for amino acids in the BIODYN2 soil, no chemoattraction can be expected in this case. Similarly, high concentrations of acetate and lactate were detectable in the rhizosphere and in soil without root contact (Table 3B and Supplementary Table 3B), suggesting that these compounds are rather products of organic carbon turnover than directly released from plant roots.

### Rhizosphere Microbiota and Stress-Related Gene Expression of the Host Plant

The rhizosphere of lettuce grown in HU-org and BIODYN2 was enriched in fungal pathotrophs and patho-saprotrophs (Supplementary Table 5). This could be a possible reason for a significantly higher expression of jasmonic acid signaling-dependent genes (*PDF1.2*) and lipoxigenase (*LOX*) genes in the shoot tissue of plants grown in soils with organic vs. mineral fertilization, as previously reported also by Chowdhury et al. (2019). Recent findings suggest that the rhizosphere microbiota act as an additional immune barrier for plants and can activate long distance signaling pathways, involving ethylene, salicylic and jasmonic acid, which led to systemic resistance in distal tissues (Spoel and Dong, 2012; Hacquard et al., 2017). The *RbohD* gene, encodes NADPH oxidase (respiratory burst oxidase), involved in free radical (ROS) production for pathogen defense after contact with pathogen-associated molecular patterns (PAMPs) and has also been reported as systemic response to elevated pathogen abundance in the root/rhizosphere (Miura and Tada, 2014; Kadota et al., 2015). Our results showed that this gene was upregulated in both soils with organic fertilization (BIODYN2, HU-org) including high relative abundances of potential pathotrophs (Supplementary Table 5).

Redistribution of iron upon pathogen attack is another defense response in various plant species, leading to accumulation of Fe^3+^ in the apoplast of epidermal cells, where it induces the production of H_2_O_2_, resulting in a defensive oxidative burst (Verbon et al., 2017). This can induce local iron deficiencies in the cytosol (Liu et al., 2007) and may explain the preferential upregulation of the *OPT3* gene in lettuce plants grown in organic HU-org and BIODYN2 soils (Figure 2). The gene encodes an oligopeptide transporter involved in phloem-mediated Fe transport, induced under conditions of Fe deficiency (Stacey et al., 2008). Moreover, pathogen infection can induce systemic activation of genes involved in root-induced Fe acquisition (*FRO2*, *IRT1*; Verbon et al., 2017) and may offer an explanation for the particularly high shoot Fe concentration in plants, grown in DOK-LTE soils (Table 2), associated with higher pathogen (*Olpidium*) enrichment in the rhizosphere compared to HUB-LTE soils (Supplementary Table 4B). Interestingly, the expression of the nitrate reductase gene (*NIA1*) was significantly increased in lettuce plants in soils with long-term organic fertilization history. This may be attributed to a higher N mineralization potential because of long-term manure-based fertilization in BIODYN2 and HU-org soils. However, in the experimental setup, all treatments received full N supply via nitrate fertilization, sufficient for the duration of our experiment, without any treatment differences of the N nutritional status (Table 2). An alternative explanation may be provided by the results of Vujinović et al. (2020), demonstrating superior induction of nitrate reductase genes in maize by humic substances isolated from soils with long-term organic fertilization as compared with conventional mineral fertilization.

Plant defense mechanisms are complex and in soil culture where the roots are in continuous contact with diverse microbes, it is difficult to assign a direct systemic effect of particular microbial taxa to changes in the gene expression in the shoots. The generally higher expression of stress-related genes recorded in the shoots of organically grown plants are interesting indicators for a systemic defense response. However, we were not able to detect any specific response in stress gene expression to the particularly intense infection with the root pathogen *Olpidium*, affecting plants grown in BIODYN2 soil. Severe growth depressions and infection symptoms suggest that the upregulation of the respective defense genes alone was not sufficient to counteract the pathogen attack. Certain pathogens are able to overcome these primary defense lines via specific elicitors with potential to inhibit signaling pathways or the synthesis and accumulation of defense compounds by the host plant (Henry et al., 2012; Fitzpatrick et al., 2020). This scenario may be reflected by the reduced accumulation of the antifungal root exudate benzoate in the rhizosphere of the *Olpidium*-infected plants grown in BIODYN2 soil (Table 3B). Comparison with plants in CONMIN soil, lacking disease symptoms despite a similar relative rhizosphere abundance of the *Olpidium* pathogen and even lower systemic expression of defense genes, suggest that additional factors are required for pathogen suppression. These factors may comprise the recruitment of beneficial microorganisms (e.g., *Pseudomonas* sp., *Mortierella elongata*) via specific root exudate patterns (i.e., increased levels of succinate, malate, glucose), indicated by a reduction in stress gene expression as part of a “cry for help” strategy employed by the host plant (Fitzpatrick et al., 2020; Liu et al., 2020). Additionally, the increased production of root exudates with antifungal potential (i.e., benzoate) was stimulated by the presence of beneficial microbes (Windisch et al., 2017). A chronological investigation of the plant responses to pathogen infection and a more comprehensive characterization of metabolic stress indicators could reveal more evidence in this concern.

## Conclusion

The present study confirmed the well-documented selective effects of the soil site and the rhizosphere as important determinants shaping the diversity and composition of soil microbial communities. However, with respect to the impact of fertilization management, frequently reported benefits of long-term organic fertilization, such as increased soil organic matter, increased microbial biomass and metabolic activity, species richness and higher abundance of plant beneficial microorganisms were only partially confirmed. A significant increase of soil organic matter was only detectable for BIODYN2 soil. The rhizosphere microbial diversity under long-term organic fertilization was not consistently increased, for both bacterial/archaeal and fungal communities. Beneficial effects on the relative abundance of plant growth-promoting microorganisms were only detectable for AMF but not generally for potential plant growth-promoting microorganisms (*Pseudomonas*, *Mortierella*), and were sometimes (BIODYN2) even associated with severe growth depressions likely caused by the lettuce pathogen *Olpidium*. A consistently increased systemic expression of stress-related genes in shoot tissue of plants growing in soils with long-term organic fertilization, previously interpreted as stress-priming effect (Chowdhury et al., 2019), was not associated with suppression of *Olpidium*. In contrast, increased rhizosphere relative abundance of *Pseudomonas* sp. and *M. elongata* and increased accumulation of the antifungal root exudate benzoic acid coincided with reduction in disease severity caused by *Olpidium* in the CONMIN soil with long-term mineral fertilization, suggesting a function in pathogen defense. Obviously, the characteristics reported for long-term organic fertilization cannot be generalized as similarly documented in previous studies (Hartman et al., 2018; Chowdhury et al., 2019).

Furthermore, the results suggest that the effects of fertilization management on the chemical composition of the rhizosphere soil solution, as a major driver for shaping rhizosphere microbial communities, are not simply controlled by root activity and rhizodeposition but also by site-specific factors e.g., turn-over of soil organic matter and substrate competitions between different groups of rhizosphere microorganisms. Additionally, soil properties such as moisture level and water-holding capacity or the availability of specific mineral nutrients may act as modulators for the assemblage of rhizosphere microbiota.

**FIGURE 8:**
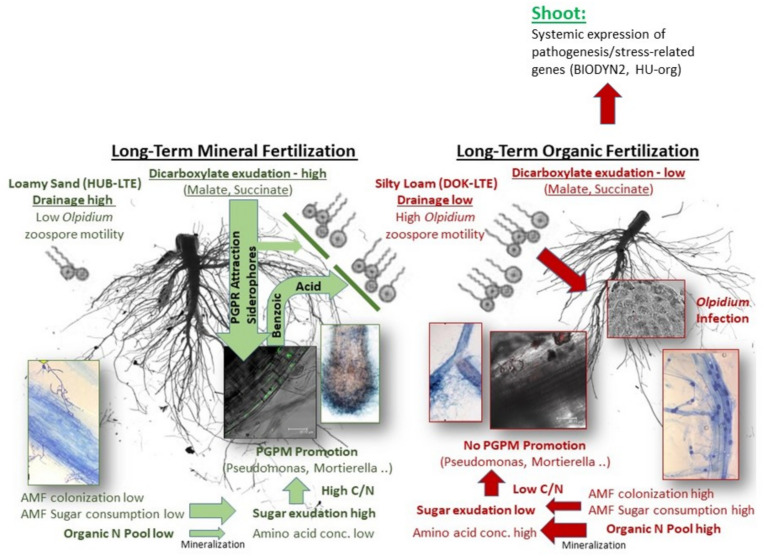
Hypothetic model, summarizing the potential interplay between fertilization management, soil factors, root exudation and rhizosphere microbial communities in lettuce-*Olpidium* interactions. Pathogen-conducive scenarios are indicated by red labels, while green labels represent pathogen-suppressive scenarios. A high drainage potential of the loamy sand soils (HUB-LTE) counteracts the motility of *Olpidium* zoospores, which is stimulated by low moisture draining in the silty loam soils (DOK-LTE), thereby promoting *Olpidium* pathogen pressure. In the pathogen suppressive scenario, long-term mineral fertilization reduces the soil AMF inoculum (arbuscular mycorrhizal fungi). Consequently, lower AMF colonization is associated with lower sugar consumption by the fungal partner and with higher sugar exudation. The higher availability of easily accessible carbohydrates attracts rhizosphere colonization by Gammaproteobacteria (i.e., *Pseudomonadaceae*) and *Mortierella* with documented plant growth-promoting and pathogen-suppressive properties. This is further promoted by higher root exudation of dicarboxylates (i.e., succinate) with known functions as chemoattractants and substrates for siderophore production in many *Pseudomonas* species with plant growth-promoting properties. Moreover, *Pseudomonas* inoculation can stimulate root exudation of benzoate with antifungal properties in lettuce. By contrast, in the pathogen-conducive scenario, high relative abundance of AMF in the rhizosphere associated with high fungal sugar consumption drastically reduces the rhizosphere sugar concentrations of lettuce plants grown in DOK-LTE soils with long-term organic fertilization (BIODYN2), counteracting root colonization by potentially beneficial *Pseudomonadaceae* and *Mortierella* species, thereby favoring *Olpidium* infection. A low C/N ratio in the rhizosphere soil solution, promoted by high background concentrations of amino acids probably related with more intense organic N mineralization under long-term organic fertilization further reduces the relative abundance of *Pseudomonadaceae* in the rhizosphere (for details, see section “Discussion”). Photos by courtesy of Markus Weinmann, Namis Eltlbany, and Khalid Hameed.

The results point to a complex network of belowground interactions between plant roots, physicochemical soil properties and different groups of rhizosphere microbiota (schematically summarized in Figure 8), with an important role in shaping also the aboveground characteristics of the plants.

Although the presented results, so far based on model experiments under controlled conditions are mainly based on descriptions of coinciding relationships without consideration of temporal or spatial variations and lacking detailed mechanistic explanations, the study can provide a starting point for more focused investigations into the related processes. The identification of key players in these networks and their effects on plant health and crop performance may provide important information to optimize the interactive effects of fertilization management, related to rhizosphere processes and development of novel plant growth-promoting microorganisms inoculants. Finally, our results may contribute to the development of practical approaches according to the concept of “soil biological engineering” (Bender et al., 2016).

## Data Availability Statement

The datasets presented in this study can be found in online repositories. The names of the repository/repositories and accession number(s) can be found below: https://www.ncbi.nlm.nih.gov/, PRJNA622892 and http://www.ebi.ac.uk/ena/data/view/PRJEB39853.

## Author Contributions

SW, LS, DB, GN, RG, JG, and KS conceived and designed the experiment. SW, LS, DB, and SC conducted the experiments and collected the data. SW, LS, and DB wrote the manuscript with GN, UL, RG, JG, and KS. FW, BH, NM, and SW developed and performed the UHPLC-MS analysis of amino acids and benzoic acid. WA and SW developed and performed the HPLC-ELSD analysis of sugars. SW and GN developed and performed the RP-HPLC analysis of organic acids. DB, KS, JN, and SS performed and evaluated the sequencing of bacterial and archaeal communities. LS, IS, and JG developed and evaluated the sequencing and analysis of fungal communities. SC and MR developed and performed the plant gene expression analysis. SW and AE performed the detection of pathogen infection in the root tissue. All authors approved the final manuscript.

## Conflict of Interest

The authors declare that the research was conducted in the absence of any commercial or financial relationships that could be construed as a potential conflict of interest.

## Footnotes

1 www.arabidopsis.org

2 https://www.ncbi.nlm.nih.gov/sra

3 https://www.mothur.org/wiki/RDP_reference_files

4 https://www.ebi.ac.uk/ena/browser/home

5 http://hannonlab.cshl.edu/fastx_toolkit/

6 https://galaxyproject.org

## Supplementary Material

Supplementary Figure 1 Habitus of lettuce plants grown for six weeks in BIODYN2 soil infected with *Olpidium* sp. (likely *Olpidium brassicae*) **(A–C)** infected fine roots with intracellular fungal structures (arrows) and inhibition of root hair development, **(D)** *Olpidium* resting spore ín the root tissue **(E)** sporangium of *Olpidium* in the root tissue. Photos by courtesy of Abbas El-Hasan.

Supplementary Table 1 List of plant genes selected for expression analysis with their corresponding loci, functions in *A. thaliana* and primer sequences.

Supplementary Table 2 Shoot and root biomass production and root growth parameters of lettuce (cv. Tizian). The plants were grown in minirhizotron culture in two independent experiments during a culture period of nine weeks and six weeks, respectively, on soils with long-term organic (HU-org, BIODYN2) or mineral (HU-min, CONMIN) fertilization history. Means ± standard errors of four independent replicates per treatment. Different lowercase letters indicate significant differences between organic vs. mineral fertilization tested separately for the sites DOK-LTE and HUB-LTE by one-way ANOVA, Tukey’s HSD pairwise test, *p* ≤ 0.05.

Supplementary Table 3 Sugars (A), carboxylates (B) and amino acids (C) in soil solutions of samples collected without visible contact to lettuce roots grown in soils with long-term organic (HU-org, BIODYN2) or mineral (HU-min, CONMIN) fertilization history. The plants were grown in minirhizotron culture for nine weeks. Exudate collection was undertaken by micro-sampling with sorption filters on bulk soil, without any root contact. Means ± standard errors. Different lowercase letters indicate significant differences between organic vs. mineral fertilization tested separately for each long-term experimental site DOK-LTE and HUB-LTE by t-test (p ≤ 0.05). n.d. = not detectable.

Supplementary Table 4 Taxonomic composition of (A) bacterial/archaeal phyla and proteobacterial classes and (B) fungal phyla represented as relative abundances in root-associated soil and rhizosphere of lettuce (cv. Tizian). The plants were grown in soils under long-term organic (HU-org, BIODYN2) or mineral (HU-min, CONMIN) fertilization. Different lowercase letters indicate significant differences between organic vs. mineral fertilization tested separately for each long-term experimental site and habitat by edgeR (FDR < 0.05). Means of relative abundance ± standard errors.

Supplementary Table 5 Ecological assignment of fungal genera represented as relative abundance with FUNGuild in root-associated soils and rhizosphere of lettuce (cv. Tizian) grown in soils of DOK-LTE (BIODYN2 vs. CONMIN) and HUB-LTE (HU-org vs. HU-min). Different lowercase letters indicate significant differences (FDR < 0.05) of represented genera between organic vs. mineral fertilization tested separately for each long-term experimental site and habitat based on the results of edgeR analyses. Means of relative abundance ± standard errors. Relative abundances (> 0.5%) with significant differences are marked in bold.

Supplementary Table 6 Fungal taxa in the root-associated soils of lettuce (cv. Tizian) differing significantly (FDR < 0.05) in relative abundance depending on long-term organic vs. mineral fertilization practice at HUB-LTE (HU-org vs. HU-min) (A) and DOK-LTE (BIODYN2 vs. CONMIN) (B). Only taxa with > 1.0% relative abundance are displayed. For OTUs on species level the similarity compared to the database are represented. Means of relative abundance ± standard errors. Bold numbers indicate significant enrichment.

Supplementary Text 1 Materials and Methods description of microbial community analyses: Bacterial and archaeal communities.

Supplementary Text 2 Materials and Methods description of microbial community analyses: Fungal communities.
