# Supplementary material for: Impact of Long-Term Organic and Mineral Fertilization on Rhizosphere Metabolites, Root–Microbial Interactions and Plant Health of Lettuce
Source: Front Microbiol. 2021 Jan 13;11:597745. doi: 10.3389/fmicb.2020.597745 (PMC7838544; doi:10.3389/fmicb.2020.597745)
Supplement: Supplementary Figure 1 — Habitus of lettuce plants grown for six weeks in BIODYN2 soil infected with Olpidium sp. (likely Olpidium brassicae) (A–C) infected fine roots with intracellular fungal structures (arrows) and inhibition of root hair development, (D) Olpidium resting spore ín the root tissue (E) sporangium of Olpidium in the root tissue. Photos by courtesy of Abbas El-Hasan. [file Image_1.PDF]

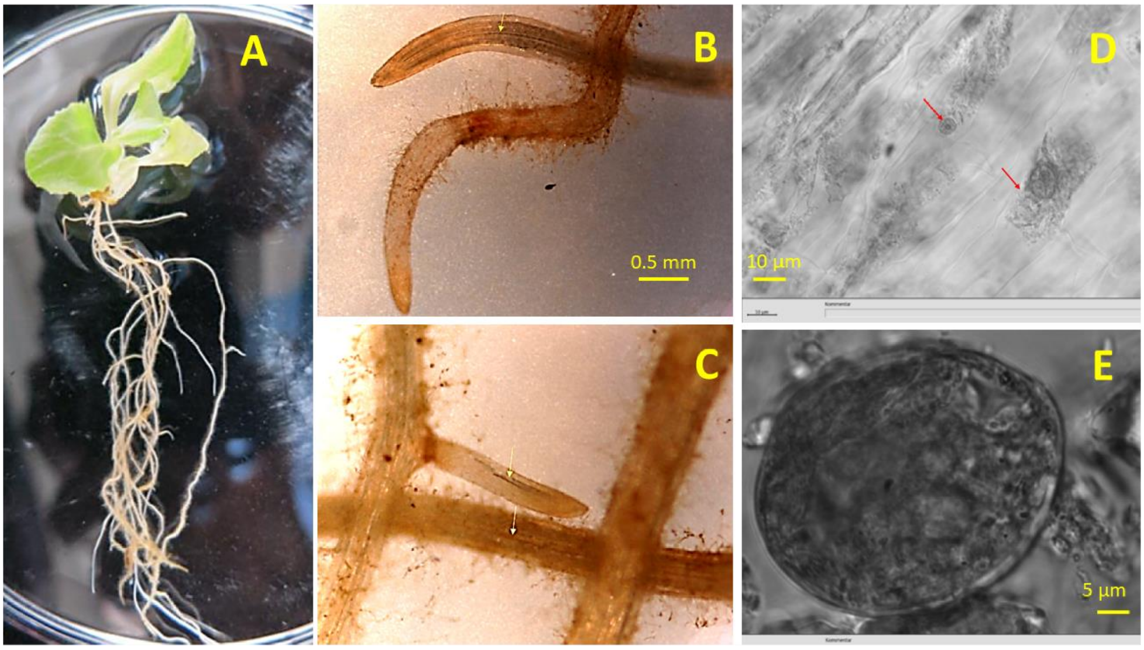

Supplementary Figure 1 | Habitus of lettuce plants grown for six weeks in BIODYN2 soil infected with *Olpidium* sp. (likely *Olpidium brassicae*) (A), (B) and (C) infected fine roots with intracellular fungal structures (arrows) and inhibition of root hair development, (D) *Olpidium* resting spore in the root tissue (E) sporangium of *Olpidium* in the root tissue. Photos by courtesy of Abbas El-Hasan.
