# Supplementary material for: Impact of Long-Term Organic and Mineral Fertilization on Rhizosphere Metabolites, Root–Microbial Interactions and Plant Health of Lettuce
Source: Front Microbiol. 2021 Jan 13;11:597745. doi: 10.3389/fmicb.2020.597745 (PMC7838544; doi:10.3389/fmicb.2020.597745)
Supplement: Supplementary Table 1 — List of plant genes selected for expression analysis with their corresponding loci, functions in A. thaliana and primer sequences. [file Data_Sheet_1.docx]

# Supplementary materials

## Tables

**Supplementary Table 1ǀ List of plant genes** selected for expression analysis with their corresponding loci, functions in *A. thaliana* and primer sequences.

| **Name of gene**  **(Locus tag in *Arabidopsis thaliana*)** | **Documented functions in *Arabidopsis thaliana*** | **Primer sequences (5‘-3‘)**  All primers were designed in this study and have an annealing temperature of 55°C |
| --- | --- | --- |
| *OPT3* (AT4G16370) | Iron transporter involved in systemic iron, zinc and cadmium distribution within the plant. | OPTf - GGCTTGTCACCGGAATGATC  OPTr - TGCAAGGCGAAGAACAACAA |
| *NIA1*  (AT1G77760) | Nitrate reductase, nitrate induced expression and involved in nitrate assimilation. | NIAf - ACCTTCACCATGTCCGAAGT  NIAr - TGAGTATGCTGTCACTGCCA |
| *PR1*  (AT2G14610) | Pathogenesis related protein 1, Salicylic acid (SA) dependent expression, involved in resistance against broad spectrum of pathogens. | PR1f - GAGAAGGCCGATTATGATTA  PR1r - ATTATTGCATTGAACCCTTG |
| *PDF1.2* (AT5G44420) | Plant defensin factor involved in Jasmonic acid (JA)/ Ethylene (Et) dependent pathogen defense responses. Involved in Induced systemic resistance (ISR). | PDF1.2f - ACAAGATATGCGAGCGGAGA  PDF1.2r - TGACAGGCTCCATGTTTTGC |
| *LOX1*  (AT1G55020) | Lipoxigenase; Upstream gene involved in the oxylipin metabolic pathway. Involved in the signaling of wounding response and JA induced defense against specific pathogens. | LOX1f - AAGAGCAGAAGCCACCCATA  LOX1r - GTGGAAGGAACTGCGAGAAG |
| *WRKY70* (AT3G56400) | Transcription factor involved in both SA- and JA-mediated signal pathways. Also involved in abiotic stress signaling. | WRKY70f - GCACACACAAAACCGACCAA  WRKY70r - AGTTGTTGCAAGTATGGTGTCC |
| *WRKY25* (AT2G30250) | Negative regulator of SA-mediated defense responses, elevated expression in response to oxidative stress, heat stress or wounding. | WRKY25f - TGTTCAATGAGGAAGAAGGTGG  WRKY25r - TCGTTTGGTGGATTGTGGTTT |
| *CAT1* (AT1G20630) | Catalase induced by hydrogen peroxide, abscisic acid (ABA), drought, and salt stress. | CAT1f - GGTCCAAGGCGATGTCTTTG  CAT1r - ATGAACAGCTGGCGTTTTGT |
| *PER50* (AT4G37520) | Peroxidase; Responses to environmental stresses such as wounding, pathogen attack and oxidative stress. | PER50f - CTGTCAACACATGGGCTTCC  PER50r - TCCCACTTCGACCCGTTTTA |
| *ERF6* (AT4G17490) | Et- Response Factor family transcription factor. Responses to oxidative stress and biotic stress induced by biotrophic and necrotrophic pathogens. | ERF6f - CAAAACGTCGCGGATCTAGG  ERF6r - GACGCAACCTCAAGTGGAAA |
| *ZAT10* (AT1G27730) | Zinc finger protein; Transcriptional repressor involved in abiotic stress responses. Positive transcriptional regulator for salinity, heat and osmotic stress. | ZATf - TCGTGACTCCTTCCACTTCC  ZATr - TAGGTGGACACAAGGCTAGC |
| *RbohD* (AT5G47910) | Respiratory burst oxidase homolog D. Involved in rapid reactive oxygen species (ROS) production on perception of pathogen-associated molecular patterns (PAMPs) by pattern recognition receptors (PRRs). | RbohDf - ACAGGGTTCTTTCGACTGGT  RbohDr - AATTAGAGCAGACCTGGCGT |
| *RbohF* (AT1G64060) | Respiratory burst oxidase homolog F. Involved in hypersensitive reaction (HR)-related cell death and interaction with intercellular ROS regulating pathogen defense responses. | RbohFf - TCATCGGCTCTAAGAAGCCC  RbohFr - TGCTCCAGATGACGATTACCT |
| *MYB15* (AT3G23250) | ABA inducible abiotic stress regulator, upregulated in cold and drought stress. | MYB15nf - AGGTGGGGTTGAAGAAAGGA  MYB15nr - CGTACCAGCTTTTGAAGGCA |

**Supplementary Table 2ǀ Shoot and root biomass production and root growth** parameters of lettuce (cv. Tizian). The plants were grown in minirhizotron culture in two independent experiments during a culture period of nine weeks and six weeks, respectively, on soils with long-term organic (HU-org, BIODYN2) or mineral (HU-min, CONMIN) fertilization history. Means ± standard errors of four independent replicates per treatment. Different lowercase letters indicate significant differences between organic vs*.* mineral fertilization tested separately for the sites DOK-LTE and HUB-LTE by one-way ANOVA, Tukey´s HSD pairwise test, *p* ≤ 0.05.

| **Fresh biomass production and root growth parameters of lettuce (cv. Tizian)** | | | | |  | | | |
| --- | --- | --- | --- | --- | --- | --- | --- | --- |
| **Plant biomass** | **DOK-LTE** | | | | **HUB-LTE** | | | |
|  | **BIODYN2** | **CONMIN** | **BIODYN2** | **CONMIN** | **HU-org** | **HU-min** | **HU-org** | **HU-min** |
|  | 9 weeks culture period | | 6 weeks culture period | | 9 weeks culture period | | 6 weeks culture period | |
| Shoot biomass [g plant^-1^] | 5.83 ± 0.89 b | 9.93 ± 1.31 a | 0.77 ± 0.09 b | 1.58 ± 0.22 a | 10.51 ± 0.82 a | 12.71 ± 0.87 a | 3.98 ± 0.48 b | 7.55 ± 0.64 a |
| Root biomass [g plant^-1^] | 0.43 ± 0.15 b | 1.93 ± 0.10 a | 0.64 ± 0.04 b | 1.06 ± 0.07 a | 0.948 ± 0.20 a | 0.87 ± 0.16 a | 3.03 ± 0.45 b | 4.94 ± 0.34 a |
| Total root length [cm] | 600.01 ± 149.42 b | 2665.42 ± 274.67 a | 56.20 ± 4.49 b | 85.66 ± 8.84 a | 1482.12 ± 466.62 a | 603.92 ± 41.1 a | 142.75 ± 25.83 a | 197.20 ± 26.95 a |
| Root hair length [mm] | Not determined | Not determined | 0.16 ± 0.03 b | 0.47 ± 0.02 a | Not determined | Not determined | 0.37 ± 0.04 a | 0.51 ± 0.08 a |

**Supplementary Table 3ǀ Sugars (A), carboxylates (B) and amino acids (C) in soil solutions** of samples collected without visible contact to lettuce roots grown in soils with long-term organic (HU-org, BIODYN2) or mineral (HU-min, CONMIN) fertilization history. The plants were grown in minirhizotron culture for nine weeks. Exudate collection was undertaken by micro-sampling with sorption filters on bulk soil, without any root contact. Means ± standard errors. Different lowercase letters indicate significant differences between organic vs. mineral fertilization tested separately for each long-term experimental site DOK-LTE and HUB-LTE by t-test (p ≤ 0.05). n.d. = not detectable.

| 1. **Sugars in the soil solution [**nmol cm^-1^ sorption filter] | | | | |
| --- | --- | --- | --- | --- |
|  | **HUB-LTE** | | **DOK-LTE** | |
|  | **HU-org** | **HU-min** | **BIODYN2** | **CONMIN** |
|  | Soil solution | | Soil solution | |
| Fructose | n.d. b | 1.47 ± 0.25 a | n.d. b | 0.82 ± 0.09 a |
| Glucose | n.d. b | 1.40 ± 0.19 a | n.d. b | 0.72 ± 0.02 a |
| Sucrose | n.d. | n.d. | n.d. b | 0.46 ± 0.09 a |
| Maltose | n.d. | n.d. | n.d. a | 0.62 ± 0.22 a |
| **Sum** | n.d. b | 2.88 a | n.d. b | - 1. a |

| 1. **Carboxylates in the soil solution [**nmol cm^-1^ sorption filter**]** | | | | |
| --- | --- | --- | --- | --- |
|  | **HUB-LTE** | | **DOK-LTE** | |
|  | **HU-org** | **HU-min** | **BIODYN2** | **CONMIN** |
|  | Soil solution | | Soil solution | |
| Malate | 12.42 ± 3.17 a | n.d. b | n.d. | n.d. |
| Citrate | 3.13 ± 0.94 a | 5.21 ± 1.06 a | 2.38 ± 0.56 a | 1.64 ± 0.29 a |
| Succinate | n.d. | n.d. | n.d. | n.d. |
| Fumarate | n.d. b | 0.28 ± 0.06 a | 0.62 ± 0.03 a | n.d. b |
| Benzoate | 0.06 ± 0.03 a | 0.03 ± 0.005 a | 0.02 ± 0.003 b | 0.09 ± 0.003 a |
| Acetate | n.d. a | 21.48 ± 9.78 a | 8.44 ± 3.65 a | 4.33 ± 0.78 a |
| Lactate | 68.54 ± 12.32 a | 70.99 ± 28.74 a | 26.17 ± 4.93 a | 10.26 ± 2.75 b |
| **Sum** | 84.17 a | 98.00 a | - 1. a | - 1. a |

| 1. **Amino acids in the soil solution [**nmol cm^-1^ sorption filter**]** | | | | |
| --- | --- | --- | --- | --- |
|  | **HUB-LTE** | | **DOK-LTE** | |
|  | **HU-org** | **HU-min** | **BIODYN2** | **CONMIN** |
|  | Soil solution | | Soil solution | |
| Glutamic acid | 0.008 ± 0.005 a | n.d. a | 0.006 ± 0.002 a | n.d. a |
| Asparagine | 0.017 ± 0.010 a | 0.042 ± 0.002 a | 1.045 ± 0.475 a | 0.004 ± 0.001 a |
| Serine | 0.046 ± 0.004 a | 0.026 ± 0.001 b | 0.395 ± 0.045 a | 0.046 ± 0.002 b |
| Glutamine | 0.012 ± 0.005 a | 0.038 ± 0.019 a | 0.107 ± 0.014 a | n.d. b |
| Glycine | 0.052 ± 0.003 a | 0.022 ± 0.001 b | 0.354 ± 0.031 a | 0.047 ± 0.002 b |
| Threonine | 0.003 ± 0.001 a | 0.005 ± 0.001 a | 0.109 ± 0.016 a | n.d. b |
| Histidine | n.d. | n.d. | 0.031 ± 0.004 a | n.d. b |
| Alanine | 0.014 ± 0.003 a | n.d. b | 0.247 ± 0.023 a | 0.008 ± 0.002 b |
| Proline | 0.006 ± 0.002 a | 0.005 ± 0.001 a | 0.062 ± 0.004 a | 0.008 ± 0.001 b |
| Cystine | 0.003 ± 0.002 a | n.d. a | n.d. | n.d. |
| Thyrosine | n.d. a | 0.005 ± 0.001 a | 0.088 ± 0.015 a | n.d. b |
| Methionine | n.d. b | 0.019 ± 0.004 a | 0.164 ± 0.011 a | n.d. b |
| Isoleucine | 0.006 ± 0.001 a | 0.005 ± 0.001 a | 0.029 ± 0.006 a | n.d. b |
| Leucine | 0.007 ± 0.001 a | 0.003 ± 0.001 b | 0.039 ± 0.009 a | n.d. b |
| Phenylalanine | 0.042 ± 0.012 a | 0.006 ± 0.001 a | 0.031 ± 0.002 a | 0.015 ± 0.001 b |
| **Sum** | 0.216 a | 0.174 a | 2.706 a | 0.126 b |

**Supplementary Table 4ǀ Taxonomic composition of (A) bacterial/archaeal phyla and proteobacterial classes and (B) fungal phyla represented as relative abundances in root-associated soil and rhizosphere** **of lettuce (cv. Tizian)**. The plants were grown in soils under long-term organic (HU-org, BIODYN2) or mineral (HU-min, CONMIN) fertilization. Different lowercase letters indicate significant differences between organic vs*.* mineral fertilization tested separately for each long-term experimental site and habitat by edgeR (FDR < 0.05). Means of relative abundance ± standard errors.

| 1. **Taxonomic composition of bacterial/archaeal communities in root-associated soil and rhizosphere of lettuce (cv. Tizian)** | | | | | | | | | |
| --- | --- | --- | --- | --- | --- | --- | --- | --- | --- |
| **Kingdom** | **Phylum/Class** | **Root-Associated soil** | | | | **Rhizosphere** | | | |
|  |  | **HUB-LTE** | | **DOK-LTE** | | **HUB-LTE** | | **DOK-LTE** | |
|  |  | **HU-org**  **[%]** | **HU-min**  **[%]** | **BIODYN2 [%]** | **CONMIN**  **[%]** | **HU-org**  **[%]** | **HU-min**  **[%]** | **BIODYN2**  **[%]** | **CONMIN**  **[%]** |
| Bacteria | Acidobacteria | 16.02 ± 0.82 a | 19.43 ± 0.23 a | 11.72 ± 0.56 b | 12.42 ± 0.47 a | 2.78 ± 0.14 a | 2.20 ± 0.42 a | 5.96 ± 0.67 a | 3.55 ± 0.60 a |
| Bacteria | Actinobacteria | 15.12 ± 0.62 a | 12.72 ± 0.39 b | 10.40 ± 0.29 b | 12.20 ± 0.42 a | 9.19 ± 2.85 a | 9.03 ± 2.28 a | 7.28 ± 0.82 b | 8.20 ± 0.78 a |
| Bacteria | Bacteria_unclassified | 8.68 ± 0.58 a | 10.74 ± 0.38 a | 8.09 ± 0.32 a | 6.95 ± 0.21 a | 2.43 ± 0.44 a | 2.35 ± 0.49 a | 6.41 ± 1.25 a | 2.69 ± 0.51 a |
| Bacteria | Bacteroidetes | 4.54 ± 0.27 a | 4.26 ± 0.28 a | 8.04 ± 0.04 a | 7.09 ± 0.19 a | 5.10 ± 0.33 a | 2.47 ± 0.51 a | 7.08 ± 0.19 a | 3.84 ± 0.95 a |
| Bacteria | Candidatus_Saccharibacteria | 1.63 ± 0.31 a | 1.45 ± 0.21 a | 0.66 ± 0.03 b | 1.07 ± 0.13 a | 3.49 ± 1.23 a | 4.93 ± 2.22 a | 2.05 ± 0.27 a | 1.57 ± 0.66 a |
| Bacteria | Chloroflexi | 1.68 ± 0.20 a | 2.65 ± 0.14 a | 1.53 ± 0.04 a | 1.26 ± 0.05 a | 0.65 ± 0.16 a | 0.79 ± 0.14 a | 1.82 ± 0.38 a | 0.74 ± 0.14 a |
| Bacteria | Cyanobacteria/Chloroplast | 0.08 ± 0.01 a | 0.09 ± 0.04 a | 0.26 ± 0.11 a | 0.69 ± 0.35 a | 0.03 ± 0.00 a | 0.03 ± 0.02 a | 1.18 ± 0.71 a | 0.04 ± 0.02 b |
| Bacteria | Firmicutes | 15.55 ± 0.68 a | 13.07 ± 0.84 b | 14.48 ± 0.39 a | 12.42 ± 0.98 a | 6.87 ± 1.08 a | 5.49 ± 1.25 a | 11.33 ± 1.03 a | 5.60 ± 1.52 a |
| Bacteria | Gemmatimonadetes | 2.64 ± 0.13 a | 3.04 ± 0.19 a | 0.43 ± 0.02 b | 0.77 ± 0.06 a | 0.37 ± 0.04 a | 0.26 ± 0.04 a | 0.22 ± 0.05 a | 0.29 ± 0.06 a |
| Bacteria | Nitrospirae | 1.02 ± 0.08 a | 0.83 ± 0.07 b | 1.67 ± 0.05 a | 1.38 ± 0.10 a | 0.16 ± 0.01 a | 0.07 ± 0.01 a | 0.62 ± 0.15 a | 0.39 ± 0.12 a |
| Bacteria | Alphaproteobacteria | 15.63 ± 0.89 a | 15.52 ± 1.18 a | 9.33 ± 0.31 b | 12.15 ± 0.40 a | 23.84 ± 2.36 a | 25.22 ± 6.80 a | 22.33 ± 1.03 a | 13.95 ± 4.25 a |
| Bacteria | Betaproteobacteria | 5.54 ± 0.33 a | 4.64 ± 0.40 b | 3.94 ± 0.29 a | 3.43 ± 0.10 a | 15.04 ± 0.80 a | 5.95 ± 2.20 a | 12.65 ± 3.55 a | 14.34 ± 3.72 a |
| Bacteria | Deltaproteobacteria | 2.52 ± 0.14 a | 2.67 ± 0.23 a | 2.62 ± 0.21 a | 2.48 ± 0.12 a | 0.81 ± 0.16 a | 0.32 ± 0.06 a | 2.34 ± 0.13 a | 0.85 ± 0.26 a |
| Bacteria | Gammaproteobacteria | 3.49 ± 0.31 a | 3.57 ± 0.31 a | 3.14 ± 0.08 a | 3.55 ± 0.19 a | 26.59 ± 5.91 a | 39.31 ± 12.73 a | 8.55 ± 1.36 b | 39.76 ± 13.39 a |
| Bacteria | Proteobacteria_unclassified | 0.58 ± 0.04 a | 0.42 ± 0.07 b | 0.36 ± 0.02 a | 0.36 ± 0.01 a | 0.52 ± 0.08 a | 0.23 ± 0.03 a | 0.67 ± 0.07 a | 0.35 ± 0.12 a |
| Archaea | Thaumarchaeota | 2.68 ± 0.20 a | 1.57 ± 0.24 b | 16.66 ± 0.96 a | 13.91 ± 0.47 a | 1.42 ± 0.36 a | 0.77 ± 0.18 a | 6.27 ± 0.98 a | 1.94 ± 0.57 b |
| Bacteria | Verrucomicrobia | 2.27 ± 0.11 a | 2.77 ± 0.24 a | 6.15 ± 0.18 b | 7.57 ± 0.19 a | 0.61 ± 0.11 a | 0.42 ± 0.05 a | 2.96 ± 0.56 a | 1.75 ± 0.34 a |
| Bacteria | Rare (< 1%) | 0.32 ± 0.01 b | 0.54 ± 0.04 a | 0.51 ± 0.04 a | 0.31 ± 0.04 b | 0.11 ± 0.01 a | 0.14 ± 0.04 a | 0.26 ± 0.01 a | - 1. ± 0.07 a |

| 1. **Taxonomic composition of fungal communities in root-associated soil and rhizosphere of lettuce (cv. Tizian)** | | | | | | | | | |
| --- | --- | --- | --- | --- | --- | --- | --- | --- | --- |
| **Kingdom** | **Phylum** | **Root-Associated soil** | | | | **Rhizosphere** | | | |
|  |  | **HUB-LTE** | | **DOK-LTE** | | **HUB-LTE** | | **DOK-LTE** | |
|  |  | **HU-org**  **[%]** | **HU-min**  **[%]** | **BIODYN2**  **[%]** | **CONMIN**  **[%]** | **HU-org**  **[%]** | **HU-min**  **[%]** | **BIODYN2**  **[%]** | **CONMIN**  **[%]** |
| Eukaryota | Ascomycota | 68.23 ± 1.21 a | 58.43 ± 4.65 a | 21.98 ± 1.22 a | 16.67 ± 2.13 a | 62.72 ± 3.73 a | 37.17 ± 5.27 a | 5.51 ± 2.31 a | 3.41 ± 1.35 a |
| Eukaryota | Basidiomycota | 11.52 ± 0.75 b | 16.98 ± 2.75 a | 8.19 ± 0.65 b | 16.51 ± 1.37 a | 9.46 ± 0.54 a | 10.12 ± 1.01 a | 0.96 ± 0.41 a | 3.13 ± 1.49 a |
| Eukaryota | Chytridiomycota | 0.83 ± 0.33 a | 0.22 ± 0.07 b | 1.89 ± 0.27 a | 2.38 ± 1.14 a | 0.69 ± 0.17 a | 0.08 ± 0.03 b | 0.45 ± 0.27 a | 0.15 ± 0.12 a |
| Eukaryota | Entomophthoromycota | 0 ± 0 a | 0 ± 0 a | 0 ± 0 a | 0.01 ± 0 a | 0 ± 0 a | 0 ± 0 a | 0 ± 0 a | 0 ± 0 a |
| Eukaryota | Glomeromycota | 0.18 ± 0.08 a | 0.01 ± 0 b | 1.74 ± 0.28 a | 2.23 ± 0.23 a | 1.79 ± 1.13 a | 0.01 ± 0 b | 1.53 ± 0.89 a | 0.16 ± 0.03 b |
| Eukaryota | Kickxellomycota | 0 ± 0 a | 0 ± 0 a | 0 ± 0 a | 0 ± 0 a | 0 ± 0 a | 0 ± 0 a | 0 ± 0 a | 0 ± 0 a |
| Eukaryota | Mortierellomycota | 17.28 ± 1.41 a | 18.09 ± 1.75 a | 63.45 ± 0.68 a | 55.69 ± 1.89 a | 20.01 ± 3.1 b | 47.8 ± 4.99 a | 1.64 ± 0.28 b | 17.33 ± 10.96 a |
| Eukaryota | Mucoromycota | 1.65 ± 0.06 b | 5.80 ± 0.81 a | 1.51 ± 0.12 a | 1.74 ± 0.09 a | 1.01 ± 0.24 b | 4.32 ± 0.54 a | 0.05 ± 0.02 a | 0.17 ± 0.10 a |
| Eukaryota | Olpidiomycota | 0.05 ± 0.01 a | 0.01 ± 0 a | 1.00 ± 0.07 b | 4.67 ± 3.57 a | 3.97 ± 3.33 a | 0.48 ± 0.11 b | 89.84 ± 2.77 a | 75.62 ± 10.58 a |
| Eukaryota | unidentified | 0.28 ± 0.07 a | 0.47 ± 0.41 a | 0.23 ± 0.02 a | 0.10 ± 0.01 a | 0.33 ± 0.04 a | 0.03 ± 0.01 b | 0.02 ± 0.01 a | 0.03 ± 0.02 a |

**Supplementary Table 5ǀ Ecological assignment of fungal genera represented as relative abundance with FUNGuild** in root-associated soils and rhizosphere of lettuce (cv. Tizian) grown in soils of DOK-LTE (BIODYN2 vs*.* CONMIN) and HUB-LTE (HU-org vs*.* HU-min). Different lowercase letters indicate significant differences (FDR < 0.05) of represented genera between organic vs. mineral fertilization tested separately for each long-term experimental site and habitat based on the results of edgeR analyses. Means of relative abundance ± standard errors. Relative abundances (>0.5%) with significant differences are marked in bold.

| **Genus** | **Trophic Mode and Guilds** | **Root-Associated Soil** | | | | **Rhizosphere** | | | | |
| --- | --- | --- | --- | --- | --- | --- | --- | --- | --- | --- |
|  |  | **HUB-LTE** | | **DOK-LTE** | | | **HUB-LTE** | | **DOK-LTE** | |
|  |  | **HU-org**  **[%]** | **HU-min**  **[%]** | **BIODYN2**  **[%]** | **CONMIN**  **[%]** | | **HU-org**  **[%]** | **HU-min**  **[%]** | **BIODYN2**  **[%]** | **CONMIN**  **[%]** |
|  | **Saprotroph** | **9.3** | **6.5** | **6.5** | **7.8** | | **6.2** | **7.6** | **0.7** | **1.0** |
| *Arthrobotrys* | Undefined Saprotroph | 0.4 a | 0 b | 0.1 a | 0.1 a | | **1.8 a** | **0.1 b** | 0 a | 0 a |
| *Cercophora* | Dung Saprotroph | **2.6 a** | **0.1 b** | 0 a | 0 a | | 0.3 a | 0 b | 0 a | 0 a |
| *Humicola* | Undefined Saprotroph-Wood Saprotroph | **1.9 a** | **0.3 b** | 0.1 a | 0.3 a | | 1.6 a | 1.0 a | 0.1 a | 0.1 a |
| *Leucosporidium* | Soil Saprotroph-Undefined Saprotroph | 0 b | 0.1 a | 0 a | 0 a | | **0.1 b** | **0.9 a** | 0 a | 0.1 a |
| *Mucor* | Undefined Saprotroph | 0.2 a | 0.2 a | 0.4 a | 0.5 a | | **0.2 b** | **1.0 a** | 0 a | 0.1 a |
| *Nigrospora* | Undefined Saprotroph | 0 b | 0.2 a | 0 a | 0 a | | **0 b** | **0.5 a** | 0 a | 0 a |
| *Plenodomus* | Undefined Saprotroph | 0.1 a | 0.1 a | 0 b | 0.1 a | | **0.5 a** | **0.1 b** | 0 a | 0.1 a |
| *Umbelopsis* | Undefined Saprotroph | 0.3 b | 2.6 a | 0 a | 0 a | | **0.2 b** | **1.4 a** | 0 a | 0 a |
|  | **Symbiotroph** | **0.3** | **0** | **1.3** | **1.9** | | **1.7** | **0** | **1.3** | **0.1** |
| *Claroideoglomus* | Arbuscular Mycorrhizal | 0.1 a | 0 b | 0.2 a | 0.2 a | | **0.7 a** | **0 b** | 0.2 a | 0 a |
| *Funneliformis* | Arbuscular Mycorrhizal | 0.1 a | 0 b | 1.0 a | 0.8 a | | **0.9 a** | **0 b** | 1.0 a | 0.1 a |
| *Rhizophagus* | Arbuscular Mycorrhizal | 0 a | 0 a | **0 b** | **0.7 a** | | 0 a | 0 b | 0 a | 0 a |
|  | **Saprotroph-Symbiotroph** | **17.9** | **19.1** | **63.9** | **56.2** | | **20.3** | **48.7** | **1.8** | **17.5** |
| *Mortierella* | Endophyte-Litter Saprotroph-Soil Saprotroph-Undefined Saprotroph | 17.3 a | 18.1 a | 63.4 a | 55.7 a | | **20.0 b** | **47.6 a** | 1.6 a | 17.3 a |
| *Podospora* | Dung Saprotroph-Endophyte-Litter Saprotroph-Undefined Saprotroph | 0.5 a | 0.8 a | 0.4 a | 0.5 a | | **0.2 b** | **1.1 a** | 0.2 a | 0.2 a |
|  | **Pathotroph** | **3.2** | **1.3** | **2.3** | **5.7** | | **8.2** | **2.1** | **91.3** | **75.9** |
| *Ascochyta* | Plant Pathogen | 0.5 a | 0.1 b | 0.2 a | 0.2 a | | **0.5 a** | **0 b** | 0.1 a | 0 a |
| *Lectera* | Plant Pathogen | **0.6 a** | **0 b** | 0.4 a | 0.1 b | | 0.2 a | 0 b | 0 a | 0 a |
| *Moesziomyces* | Plant Pathogen | 0.3 a | 0 b | 0 a | 0 a | | **1.2 a** | **0 b** | 0 a | 0 a |
| *Olpidium* | Plant Pathogen | 0 a | 0 b | **1.0 b** | **4.7 a** | | **4.0 a** | **0.5 b** | 89.8 a | 75.6 a |
|  | **Pathotroph-Saprotroph** | **9.6** | **20.5** | **2.1** | **4.0** | | **7.2** | **9.5** | **1.4** | **0.7** |
| *Didymella* | Animal Pathogen-Plant Pathogen-Undefined Saprotroph | **2.0 a** | **0 b** | 0 a | 0 a | | **1.4 a** | **0 b** | 0 a | 0 a |
| *Exophiala* | Animal Pathogen-Undefined Saprotroph | **6.2 b** | **17.2 a** | 1.0 a | 2.2 a | | 4.9 a | 7.3 a | 1.3 a | 0.6 a |
| *Mycena* | Leaf Saprotroph-Plant Pathogen-Undefined Saprotroph-Wood Saprotroph | 0 a | 0 a | **0 b** | **0.6 a** | | 0 a | 0 a | 0 a | 0 a |
| *Rhizopus* | Plant Pathogen-Undefined Saprotroph | **0.9 b** | **3.0 a** | 0.8 a | 0.9 a | | **0.5 b** | **1.9 a** | 0 a | 0 a |
|  | **Pathotroph-Saprotroph-Symbiotroph** | **5.0** | **8.6** | **4.4** | **1.6** | | **5.8** | **4.9** | **0.9** | **0.6** |
| *Chaetomium* | Animal Pathogen-Dung Saprotroph-Endophyte-Epiphyte-Plant Saprotroph-Wood Saprotroph | 3.3 a | 5.6 a | **3.6 a** | **0.9 b** | | 4.0 a | 2.8 a | 0.7 a | 0.5 a |
| *Trichoderma* | Endophyte-Epiphyt-Fungal Parasite-Plant Pathogen-Wood Saprotroph | **0.1 b** | **0.9 a** | 0.1 a | 0.2 a | | 0 b | 0.2 a | 0 a | 0 a |
|  | **not classified or identified on genus level** | **54.6** | **43.9** | **19.5** | **22.7** | | **50.2** | **27.2** | **2.6** | **4.0** |
| *Cyberlindnera* |  | 0 a | 0 a | **0.6 a** | **0.1 b** | | 0 a | 0 a | 0 a | 0.1 a |
| *Saitozyma* |  | 2.3 a | 4.2 a | **0.1 b** | **1.9 a** | | 1.5 a | 2.6 a | 0 a | 0.3 a |
| *Slooffia* |  | 0 a | 0 a | **0 b** | **1.3 a** | | 0 a | 0 a | 0 b | 0.1 a |

**Supplementary Table 6ǀ Fungal taxa** in the **root-associated soils** **of lettuce (cv. Tizian)** differing significantly (FDR < 0.05) in relative abundance depending on long-term organic *vs.* mineral fertilization practice at HUB-LTE (HU-org vs*.* HU-min) **(A)** and DOK-LTE (BIODYN2 vs*.* CONMIN) **(B)**. Only taxa with > 1.0% relative abundance are displayed. For OTUs on species level the similarity compared to the database are represented. Means of relative abundance ± standard errors. Bold numbers indicate significant enrichment.

| 1. **Fungal taxa in root-associated soil differing significantly (FDR<0.05) in relative abundance in lettuce grown in long-term organically vs*.* minerally fertilized soils from HUB-LTE** | | | | | | | | |
| --- | --- | --- | --- | --- | --- | --- | --- | --- |
| **Phylum** | **Class** | **Order** | **Family** | **Genus** | | **OTU** | **HU-org**  **[%]** | **HU-min**  **[%]** |
| Ascomycota | Dothideomycetes |  |  |  |  | | **29.0 ± 4.5** | 3.9 ± 0.6 |
| Ascomycota | Dothideomycetes | Pleosporales |  |  |  | | **28.9 ± 4.4** | 3.8 ± 0.6 |
| Ascomycota | Dothideomycetes | Pleosporales | *Didymellaceae* |  |  | | **27.1 ± 4.3** | 2.6 ± 0.4 |
| Ascomycota | Dothideomycetes | Pleosporales | *Didymellaceae* | *Didymella* |  | | **2.0 ± 0.4** | 0 ± 0 |
| Ascomycota | Dothideomycetes | Pleosporales | *Didymellaceae* | *Didymella* | *Didymella protuberans* (100%) | | **2.0 ± 0.4** | 0 ± 0 |
| Ascomycota | Dothideomycetes | Pleosporales | *Didymellaceae* | unidentified | *Didymellaceae* sp | | **24.5 ± 4.2** | 2.5 ± 0.4 |
| Ascomycota | Sordariomycetes | Sordariales | *Lasiosphaeriaceae* | *Cercophora* |  | | **2.6 ± 0.9** | 0.1 ± 0 |
| Ascomycota | Sordariomycetes | Sordariales | *Lasiosphaeriaceae* | *Cercophora* | *Cercophora samala* (100%) | | **2.3 ± 0.8** | 0.1 ± 0 |
| Ascomycota | Sordariomycetes | Sordariales | *Chaetomiaceae* | *Humicola* |  | | **1.9 ± 0.1** | 0.3 ± 0 |
| Ascomycota | Sordariomycetes | Sordariales | *Chaetomiaceae* | *Humicola* | *Humicola grisea* (100%) | | **1.9 ± 0.1** | 0.3 ± 0 |
| Ascomycota | unidentified | unidentified | unidentified | unidentified | *Ascomycota* sp | | **1.9 ± 0.5** | 0.2 ± 0 |
| Mortierellomycota | Mortierellomycetes | Mortierellales | *Mortierellaceae* | *Mortierella* | *Mortierella minutissima* (100%) | | **1.4 ± 0.1** | 0.2 ± 0 |
| Ascomycota | Eurotiomycetes |  |  |  |  | | 8.6 ± 0.5 | **21.0 ± 0.6** |
| Ascomycota | Eurotiomycetes | Chaetothyriales |  |  |  | | 8.6 ± 0.5 | **20.9 ± 0.6** |
| Ascomycota | Eurotiomycetes | Chaetothyriales | *Herpotrichiellaceae* | *Exophiala* |  | | 6.2 ± 0.4 | **17.2 ± 0.6** |
| Ascomycota | Sordariomycetes | unidentified | unidentified | unidentified | *Sordariomycetes* sp | | 0.3 ± 0.1 | **6.2 ± 5.3** |
| Basidiomycota |  |  |  |  |  | | 11.5 ± 0.8 | **17.0 ± 2.7** |
| Basidiomycota | Agaricomycetes | Cantharellales |  |  |  | | 0.8 ± 0.2 | **3.5 ± 2.1** |
| Mucoromycota |  |  |  |  |  | | 1.6 ± 0.1 | **5.8 ± 0.8** |
| Mucoromycota | Mucoromycetes | Mucorales | *Rhizopodaceae* | *Rhizopus* |  | | 0.9 ± 0 | **3.0 ± 0.4** |
| Mucoromycota | Umbelopsidomycetes |  |  |  |  | | 0.3 ± 0 | **2.6 ± 0.5** |
| Mucoromycota | Umbelopsidomycetes | Umbelopsidales |  |  |  | | 0.3 ± 0 | **2.6 ± 0.5** |
| Mucoromycota | Umbelopsidomycetes | Umbelopsidales | *Umbelopsidaceae* |  |  | | 0.3 ± 0 | **2.6 ± 0.5** |
| Mucoromycota | Umbelopsidomycetes | Umbelopsidales | *Umbelopsidaceae* | *Umbelopsis* |  | | 0.3 ± 0 | **2.6 ± 0.5** |
| Mucoromycota | Umbelopsidomycetes | Umbelopsidales | *Umbelopsidaceae* | *Umbelopsis* | *Umbelopsis* sp | | 0.3 ± 0 | **2.6 ± 0.5** |
|  |  |  |  |  |  | |  |  |
| 1. **Fungal taxa in root-associated soil differing significantly (FDR<0.05) in relative abundance in lettuce grown in long-term organically vs. minerally fertilized soils from DOK-LTE** | | | | | | | | |
| **Phylum** | **Class** | **Order** | **Family** | **Genus** | **OTU** | | **BIODYN2**  **[%]** | **CONMIN**  **[%]** |
| Ascomycota | Sordariomycetes |  |  |  |  | | **11.0 ± 1.4** | 6.0 ± 0.8 |
| Ascomycota | Sordariomycetes | Sordariales |  |  |  | | **6.4 ± 1.9** | 2.2 ± 0.3 |
| Ascomycota | Sordariomycetes | Sordariales | *Chaetomiaceae* | *Chaetomium* |  | | **3.6 ± 2.3** | 0.9 ± 0.2 |
| Basidiomycota |  |  |  |  |  | | 8.2 ± 0.6 | **16.5 ± 1.4** |
| Basidiomycota | Microbotryomycetes |  |  |  |  | | 0 ± 0 | **1.3 ± 0.3** |
| Basidiomycota | Microbotryomycetes | Microbotryomycetes ord Incertae sedis |  |  |  | | 0 ± 0 | **1.3 ± 0.3** |
| Basidiomycota | Microbotryomycetes | Microbotryomycetes ord Incertae sedis | *Chrysozymaceae* |  |  | | 0 ± 0 | **1.3 ± 0.3** |
| Basidiomycota | Microbotryomycetes | Microbotryomycetes ord Incertae sedis | *Chrysozymaceae* | *Slooffia* |  | | 0 ± 0 | **1.3 ± 0.3** |
| Basidiomycota | Microbotryomycetes | Microbotryomycetes ord Incertae sedis | *Chrysozymaceae* | *Slooffia* | *Slooffia cresolica* (99.6%) | | 0 ± 0 | **1.3 ± 0.3** |
| Basidiomycota | Tremellomycetes |  |  |  |  | | 5.9 ± 0.2 | **11.8 ± 1.1** |
| Basidiomycota | Tremellomycetes | Tremellales |  |  |  | | 0.1 ± 0 | **1.9 ± 0.1** |
| Basidiomycota | Tremellomycetes | Tremellales | *Trimorphomycetaceae* |  |  | | 0.1 ± 0 | **1.9 ± 0.1** |
| Basidiomycota | Tremellomycetes | Tremellales | *Trimorphomycetaceae* | *Saitozyma* |  | | 0.1 ± 0 | **1.9 ± 0.1** |
| Basidiomycota | Tremellomycetes | Tremellales | *Trimorphomycetaceae* | *Saitozyma* | *Saitozyma podzolica* (100%) | | 0.1 ± 0 | **1.9 ± 0.1** |
| Olpidiomycota |  |  |  |  |  | | 1.0 ± 0.1 | **4.7 ± 3.6** |
| Olpidiomycota | Olpidiomycetes |  |  |  |  | | 1.0 ± 0.1 | **4.7 ± 3.6** |
| Olpidiomycota | Olpidiomycetes | Olpidiales |  |  |  | | 1.0 ± 0.1 | **4.7 ± 3.6** |
| Olpidiomycota | Olpidiomycetes | Olpidiales | *Olpidiaceae* | *Olpidium* |  | | 1.0 ± 0.1 | **4.7 ± 3.6** |

## Text

**Supplementary Text 1 ǀ Materials and Methods description of microbial community analyses:** Bacterial and archaeal communities

Amplification of 16S rRNA genes was performed in 25 µl volumes containing 0.625 U Hot Start Taq Polymerase (New England Biolabs GmbH, Frankfurt am Main, Germany), 1x Standard Taq Reaction buffer, 2.5 mM MgCl_2_, 0.2 mM of each dNTP, 0.4 µM of each primer and 1 µl of target DNA. Bovine serum albumin (final concentration 0.1 mg ml^-1^) was added optionally. PCR conditions were previously described by Chowdhury et al. (2019). In a second PCR reaction step the primers additionally included Illumina specific sequencing adapters and a unique combination of sequence identifier tags for each sample. After both PCR reactions, amplicon products were purified using HighPrep™ PCR Clean Up System (AC-60500, MagBio Genomics Inc., Gaithersburg, MD, United States) using a 0.65:1 (beads:PCR reaction) volumetric ratio to remove DNA fragments below 100 bp in size. Samples were normalized using SequalPrep Normalization Plate (96) Kit (Invitrogen, Maryland, MD, United States) and pooled using 5 µl volumes of each sample. The final pool volume was concentrated by using the DNA Clean and Concentrator™-5 kit (Zymo Research, Irvine, CA, United States). The pooled library concentration was determined using the Quant-iT™ High-Sensitivity DNA Assay Kit (Life Technologies, Carlsbad, CA, United States) following the specifications of the manufacturer. Before library denaturation and sequencing, the final pool concentration was adjusted to 4 nM. Amplicon sequencing was performed on an Illumina® MiSeq® platform using Reagent Kit v2 [2x250 bp] (Illumina Inc., San Diego, CA, United States). The MiSeq Controller Software Casava 1.8 (Illumina Inc., San Diego, CA, United States) was used for sequence demultiplexing and the paired-end FASTQ output files were used for the downstream sequencing analysis. Sequence analyses were performed according to acknowledged best practice guidelines (Schöler et al., 2017; Jacquiod et al., 2018).

**Supplementary Text 2 ǀ Materials and Methods description of microbial community analyses:** Fungal communities

For the amplification of fungal ITS2 regions, three PCRs per sample were conducted with 10 ng TC-DNA at different annealing temperatures (56°C ± 2°C) and the number of cycles per PCR was restricted to 24 at the midpoint of exponential phase (Sommermann et al., 2018). Subsequently, samples with the same barcodes were mixed, purified by MinElute PCR Purification Kit (QIAGEN, Hilden, Germany) and eluted in 12 μl 10 mM Tris-HCl (pH 8.5). The concentration of each sample was checked by a Qubit ® 3.0 Fluorometer (Invitrogen, Carlsbad, CA, United States) and all amplicons were pooled in equimolar amounts. The quality control and library preparation were followed by sequencing on an Illumina® MiSeq® platform (ca. 30% of an Illumina flow cell) in paired-end mode (2x 300 bp).

REFERENCES

Chowdhury, S. P., Babin, D., Sandmann, M., Jacquiod, S., Sommermann, L., Sørensen, S. J., et al. (2019). Effect of long-term organic and mineral fertilization strategies on rhizosphere microbiota assemblage and performance of lettuce. *Environ. Microbiol.* 21, 2426–2439. doi:10.1111/1462-2920.14631.

Jacquiod, S., Cyriaque, V., Riber, L., Al-soud, W. A., Gillan, D. C., Wattiez, R., et al. (2018). Long-term industrial metal contamination unexpectedly shaped diversity and activity response of sediment microbiome. *J. Hazard. Mater.* 344, 299–307. doi:10.1016/j.jhazmat.2017.09.046.

Schöler, A., Jacquiod, S., Vestergaard, G., Schulz, S., and Schloter, M. (2017). Analysis of soil microbial communities based on amplicon sequencing of marker genes. *Biol. Fertil. Soils* 53, 485–489. doi:10.1007/s00374-017-1205-1.

Sommermann, L., Geistlinger, J., Wibberg, D., Deubel, A., Zwanzig, J., Babin, D., et al. (2018). Fungal community profiles in agricultural soils of a long-term field trial under different tillage, fertilization and crop rotation conditions analyzed by high-throughput ITS-amplicon sequencing. *PLoS One* 13, 1–32. doi:10.1371/journal.pone.0195345.
